# Dysregulation of miR-375/AEG-1 Axis by Human Papillomavirus 16/18-E6/E7 Promotes Cellular Proliferation, Migration, and Invasion in Cervical Cancer

**DOI:** 10.3389/fonc.2019.00847

**Published:** 2019-09-09

**Authors:** Sridharan Jayamohan, Maheshkumar Kannan, Rajesh Kannan Moorthy, Nirmal Rajasekaran, Hun Soon Jung, Young Kee Shin, Antony Joseph Velanganni Arockiam

**Affiliations:** ^1^Molecular Oncology Laboratory, Department of Biochemistry, School of Life Sciences, Bharathidasan University, Tiruchirappalli, India; ^2^Department of Molecular Medicine and Biopharmaceutical Sciences, Graduate School of Convergence Science and Technology, Seoul National University, Seoul, South Korea; ^3^Laboratory of Molecular Pathology and Cancer Genomics, College of Pharmacy, Seoul National University, Seoul, South Korea; ^4^Enhancedbio Inc., Seongdong-gu, South Korea

**Keywords:** human papillomavirus, miR-375, astrocyte elevated gene-1, cervical cancer, cell proliferation

## Abstract

Cervical Cancer (CC) is a highly aggressive tumor and is one of the leading causes of cancer-related deaths in women. miR-375 was shown to be significantly down-regulated in cervical cancer cells. However, the precise biological functions of miR-375 and the molecular mechanisms underlying its action in CC are largely unknown. miR-375 targets were predicted by bioinformatics target prediction tools and validated using luciferase reporter assay. Herein, we investigated the functional significance of miR-375 and its target gene in CC to identify potential new therapeutic targets. We found that miR-375 expression was significantly downregulated in CC, and astrocyte elevated gene-1 (AEG-1) was identified as a target of miR-375. Our results also showed that ectopic expression of miR-375 suppressed CC cell proliferation, migration, invasion and angiogenesis, and increased the 5-fluorouracil-induced apoptosis and cell cycle arrest *in vitro*. In contrast, inhibition of miR-375 expression significantly enhanced these functions. Furthermore, HPV - 16 E6/E7 and HPV - 18 E6/E7 significantly down-regulates miR-375 expression in CC. HPV 16/18-E6/E7/miR-375/AEG-1 axis plays an important role in the regulation of cell proliferation, migration, and invasion in CC. Therefore, targeting miR-375/AEG-1 mediated axis could serve as a potential therapeutic target for CC.

## Introduction

Cervical Cancer (CC) significantly affects the health of women worldwide, especially in developing countries like India, and currently ranks as the second leading cancer in women following breast cancer (1). Human Papillomavirus (HPV) is the major risk factor for the development of CC and HPV DNA has been found in almost all cases of CC (2). More than 100 types of HPV have been identified. HPV is classified into high-risk and low-risk groups based on their oncogenic potential. High-Risk HPV (HR-HPV) types 16 and 18 strains are associated with more than 70% of CC (3, 4). HR-HPV encodes E6 and E7 which are two key oncogenes that induce a series of signals that eventually target the tumor suppressor genes TP53 and retinoblastoma (Rb). This alters the genomic stability and cell cycle regulatory pathways, prevents apoptosis, and potentially induces cellular transformation (5, 6). HPV infection by itself is not sufficient for the development of CC. It has been reported that epigenetic alterations are also needed for triggering the multi-step carcinogenesis process (7, 8).

MicroRNAs (miRNAs) are a class of short regulatory non-coding RNAs, 22 nucleotides in length, that negatively regulates target gene expression and cellular processes such as differentiation, proliferation and apoptosis by mRNA degradation, or translational repression. The activity of the miRNAs is dependent on their complementarity with the 3′- UTR (Untranslated Region) of the target mRNA (9–11). It has been reported that miRNA expression is dysregulated in many human diseases, including cancer. However, it remains unclear whether altered miRNA expression is a cause or consequence of the pathological processes (12). Epigenetic alterations are also an important process that affects miRNA dysregulation in cancer. Earlier, miR-375 was demonstrated as a tumor suppressor and was shown to be downregulated in multiple human cancers such as hepatocellular carcinoma, lung cancer, oral cancer, gastric cancer, glioma, and CC (13–18). However, whether this mechanism contributes to the miR-375 downregulation in CC is relatively unknown.

AEG-1 (Astrocyte elevated gene-1), also known as MTDH (Metadherin) and LYRIC (Lycin-rich CEACAM-1 associate protein), was originally reported as a human immunodeficiency virus-1 (HIV-1)-inducible gene in human fetal astrocytes (19, 20). AEG-1 is frequently upregulated in multiple human malignancies, such as breast cancer, hepatocellular carcinoma, non-small cell lung cancer and prostate cancer, and is correlated with disease progression and poor clinical outcomes (21–24). AEG-1 regulates several crucial events in tumor progression, including the development of chemoresistance, invasion, migration, evasion of apoptosis, cell cycle progression, and angiogenesis (25–28). Overexpression of AEG-1 enhanced anchorage-independent growth of HeLa cells and increased their invasion and migration properties, while inhibition of AEG-1 by siRNA significantly inhibits invasion and migration of cancer cells (29).

In earlier studies, it was shown that miR-375 expression is downregulated in hepatocellular carcinoma and triple-negative breast cancer cells, enabling a positive-regulation loop that maintains AEG-1 expression (30, 31). On the other hand, few studies have investigated miRNA expression profiles, especially the miR-143/miR-145/, miR-15a/miR16 and the miR-106-363 cluster in head and neck squamous cell carcinoma (HNSCC) and cervical squamous cell carcinoma with regard to HPV infection (32). However, to date only a few studies have analyzed the miR-375 profile in CC (33). Overall, whether HPV infection contributes to miR-375 downregulation in CC is relatively unclear. In addition, there are no studies on the role of miR-375/AEG-1 axis in CC development.

Since the relationship between HPV and miR-375/AEG-1 axis is largely unexplored, this study aimed to analyze the association between HPV and miR-375/AEG-1 axis and to identify the underlying molecular mechanisms and potential therapeutic targets for CC. We showed that the miR-375 expression was significantly downregulated in CC. We also showed that the downregulation of miR-375 is mediated by HPV 16/18-E6 and E7 in CC cell lines. Besides, ectopic expression of miR-375 targets AEG-1 oncogene leading to the inhibition of cervical cancer cell proliferation, migration, and invasion.

## Materials and Methods

### Cell Lines, Cell Culture

The HEK293 cell line (non-cancer human cell line) (34–38) and cervical cancer cell lines HeLa (HPV-18), SiHa, CaSki (HPV-16), and C33A (HPV-negative) were purchased from the National Center for Cell Sciences, Pune, India, and cultured in DMEM supplemented with 10% FBS and penicillin/streptomycin antibiotics (Invitrogen, Carlsbad, CA, USA). Human umbilical vein endothelial cells (HUVECs) (Thermo Fisher Scientific, Waltham, MA, USA) were cultured in Medium 200 basal media contain with large vessel endothelial supplement (Gibco). All the cells were cultured in a humidified incubator with 5% CO_2_ at 37°C.

### Transfection of miRNA Mimic, Inhibitor, and siRNA

Cervical cancer cells were seeded onto six-well plates (1 × 10^6^), and transfection experiments were conducted after the cell reached 60% confluence. For siRNA transfection, AEG-1 siRNA 1 (referred as AEG-1 siRNA) (sense: 5′-GACACUGGAGAUGCUAAUAUU-3′, antisense: 5′-UAUUAGCAUCUCCAGUGUCUU-3′), AEG-1 siRNA 2 (sense: 5′-GGUGAAGAUAACUCUACUGUU-3′, antisense: 5′-CAGUAGAGUUAUCUUCACCUU-3′) and their negative control siRNA (SIC008) were synthesized and purchased from sigma aldrich, USA. A pool of three different siRNA for HPV 16 E6/E7 and HPV 18 E6/E7 (kindly gifted from Prof. Young Kee Shin, Research Institute of Pharmaceutical Science, Seoul National University, Seoul, Republic of Korea) (39), and for miRNA transfection, miR-375 mimic (HMI0537), miR-mimic-negative control (HMC0002), miR-375 inhibitor (HSTUD0537), and miR-inhibitor-negative control (NCSTUD001) (Sigma Aldrich, USA) were transiently transfected into cells at a concentration of 20 nM using Lipofectamine RNAiMAX Reagent (Invitrogen). The mock control cells were treated with transfection reagent alone and the cells were maintained for 48 h after transfection. Transfection efficiency was analyzed by quantitative real-time PCR (qRT-PCR).

### Bioinformatics

The miRNA target prediction algorithms miRanda, MicroCosm, Diana Micro-T, and TargetScan were used to predict miRNA-375 target gene AEG-1 as well as its target regions.

### Quantitative Real-Time PCR Analysis

Total RNA was extracted from transfected cell lines using TRIZOL reagent (Invitrogen, Carlsbad, CA, USA). cDNA was synthesized using SuperScript IV First-Strand Synthesis System (Thermo Fisher Scientific) following the manufacturer's protocol. The mature miR-375 and AEG-1 mRNA expression levels were quantified through the qRT-PCR using the Fast SYBR master mix (Thermo Fisher Scientific) on the Step One Plus Real-Time PCR system (Life Technologies, Burlington, ON, Canada). U6 snRNA for miR-375 and GAPDH for AEG-1 were used as an internal control. Melting curve analysis was performed to confirm the specificity of the PCR primers. The fold changes of miR-375 and AEG-1 mRNA levels were calculated using the 2^−ΔΔ*Ct*^ method. Primers for miR-375, AEG-1 and HPV 16/18-E6/E7 have been listed in Supplementary Tables 1, 2.

### Luciferase Activity Assay

AEG-1 3′ UTR that contains putative binding sites for the miR-375 and mutated AEG-1 3′UTR was cloned into the 3′UTR of Renilla luciferase gene in the psiCHECK-2 reporter vector (kindly gifted from Prof. Stefan Wiemann, German Cancer Research Center (DKFZ), Heidelberg, Germany, and Prof. Ozgur Sahin, Bilkent University, Turkey). HEK293T cells were transfected with combinations of wild-type or mutant type AEG-1 3′UTR-Luc reporter plasmid and mimic control, miR-375 mimic, inhibitor control and miR-375 inhibitor using Lipofectamine 2,000 and 48 h post-transfection, cells were lysed using passive lysis buffer, and Renilla luciferase activity was measured using the Dual-Luciferase Assay Kit (Promega, Madison, WI, USA).

### Transwell Migration and Invasion Assay

For transwell assay, we have used two different types of AEG-1 siRNA to validate the oncogenic role of AEG-1 in CC. Mock Control, miR mimic negative control, miR inhibitor negative control, miR-375 mimic, miR-375 Inhibitor, siRNA negative control, AEG-1 siRNA, AEG-1 siRNA 2 and HPV 16,18 E6/E7 siRNAs were transfected into cervical cancer cells and after 24 h incubation, cells were collected and seeded (2 × 10^5^) on the top of the 8 μm transwell inserts (BD Biosciences, Bedford, MA, USA) with serum-free DMEM. For invasion assay, the inner surface of the insert coated with Matrigel transwell chamber (2 mg ml^−1^, BD Biosciences) was used. DMEM with 10% FBS was added to the bottom of the transwell chamber. After 48 h incubation, non-invading cells were removed from the top of the Matrigel with a cotton swab. Invaded cells that reached the lower surface of the matrigel-coated membrane were fixed with methanol and stained with 0.1% crystal violet. The CC cells invasiveness was measured by counting in five randomly selected fields under a light microscope at 20 X magnification (Carl Zeiss). For the migration assay, the procedure was similar to the transwell invasion assay except that the inner surface of the chamber had no matrigel coating.

### Apoptosis Assay by Flow Cytometry

Cell apoptosis was detected by double staining with Alexa Fluor 488-conjugated Annexin V and Propidium Iodide (PI) using the Apoptosis Detection kit (V13241, Invitrogen, Carlsbad, CA, USA) following the manufacturer's protocol. Briefly, transfected cells were harvested and washed twice with ice cold PBS. The cell pellets were suspended in 1 X Annexin binding buffer at a concentration of 2 × 10^5^ cells ml, and then the cells were incubated with Alexa Fluor 488-conjugated Annexin V and PI for 15 min in dark. The stained cells were immediately analyzed by using a BD FACS VERSE (BD, Franklin Lakes, NJ, USA) to quantify the proportion of cells in apoptosis status. All data were analyzed with Flowjo software.

### Wound Healing Assay

CC cells were transfected with miR-375 mimic, miR-375 inhibitor, AEG-1 siRNA, and their negative controls in 12 well plates (2.5 × 10^5^ cells per well). When cells reached ~90% confluency, linear scratch wounds were created uniformly on the confluent monolayer using a 200 μl pipette tip. Immediately after wounding (time 0) and at 12 h intervals for 24 h, images were taken using FLoid Cell Imaging Station (Life Technologies, USA). The migration distance was assessed by measuring the movement of the cells into a scratched wound and the width of wound gaps was measured using ImageJ analysis.

### Cell Cycle Assay

Transfected CC cells were collected and centrifuged at 600 g for 5 min and the supernatant was removed. Cells were washed twice with ice-cold PBS and fixed with ice-cold 70% ethanol for 24 h. After incubation, cells were washed with PBS again and resuspended at a final concentration of 1 × 10^6^ cells ml^−1^ in 250 μl of PI/RNase staining solution (50 mg ml^−1^/1 mg ml^−1^). Cells were incubated in the dark at 4°C for 30 min. Samples were analyzed by FACS Calibur flow cytometry.

### Fluorescent Immunocytochemistry

CC cells were seeded in with a density of 1 × 10^5^ cells per ml on the coverslips in six-well plates. Once the cells reached 60% of confluency, they were transfected with miR-375 mimic, miR-375 inhibitor, AEG-1 siRNA and their controls. After 48 h incubation, cells were fixed with 2% paraformaldehyde for 15 min. For permeabilization, cells were incubated with 0.2% TritonX-100 in PBS for 5 min. 3% BSA in PBS was used to block the cells. For detection of AEG-1, cells were incubated with an anti-AEG-1 primary rabbit monoclonal antibody (ab124789) (diluted 1:150, Abcam, Cambridge, MA, USA) for overnight at 4°C and stained with goat anti-rabbit Alexa Fluor 647-labeled secondary antibody (ab150079—Abcam) for 1 h at room temperature. For staining of filamentous actin, cells were incubated with Alexa Fluor 488 phalloidin (Invitrogen) for 30 min at room temperature and cells were washed three times with PBS after each step of the staining. Coverslips were mounted with ProLong Gold Antifade Mountant with DAPI and fluorescence images were obtained using a fluorescence microscope (Carl Zeiss, Jena, Germany). The raw LSM images were captured and exported using ImageJ software (NIH).

### MTT Assay

For cell proliferation, miR-375 mimic, miR-375 inhibitor, AEG-1 siRNA and their negative control were transfected into CC cells. After 48 h, transfected cells (1 × 10^4^) were seeded in a 96-well plate in 100 μl of a medium. 10 μl of 5 mg ml^−1^ of MTT reagent was added to the medium, and cells were incubated for 4 h in an incubator at 37°C. Absorbance was measured at 570 nm using a microplate reader (Bio-Rad, Hercules, CA, USA) according to the manufacturer's instructions at different time intervals (0, 12, and 24 h). For chemosensitivity, following transfection of 48 h, transfected cells (5 × 10^4^) were harvested and seeded into 96-well plate. Cells were treated with the IC_50_ concentration of 5-fluorouracil. The IC_50_ values were taken from Genomics of Drug Sensitivity in Cancer Project for all CC cell lines (HeLa-72.8 μM; SiHa-787 μM; CaSki-16.8 μM; C33A-11.2 μM) (40). After incubation at 37°C for different time intervals (0, 12, and 24 h), a chemosensitivity assay was performed using the MTT assay as described in cell proliferation procedure.

### HUVECs Tube-Formation Assay

Growth factor reduced matrigel matrix (100 μl, Invitrogen) was plated in 24-well plate after thawing at 4°C overnight. The 24-well plate was then incubated at 37°C for 30 min to allow the Matrigel to polymerize. miR-375 mimic, miR-375 inhibitor, AEG-1 siRNA, AEG-1 siRNA 2 and their negative controls were transfected into HUVECs cells. After transfection, HUVECs cells (2.5 × 10^4^ per well) were seeded on Matrigel-coated 24-well plates and incubated for 16 h at 37°C, tube construction was imaged under an inverted microscope (Carl Zeiss). Tube formation was determined by measuring the total tube length of HUVECs using ACAS (Automated Cellular Analysis system) provided by Ibidi (Munich, Germany).

### Western Blotting

The human Cervical Cancer cells were lysed with RIPA lysis buffer (Santa Cruz Biotechnology, Santa Cruz, CA, USA) for 48 h after transfection. The protein concentration was measured using Lowry's method. Total proteins (50 μg per lane) were separated by using 12% SDS-polyacrylamide gel electrophoresis and then were transferred onto a nitrocellulose membrane (Bio-Rad, Hercules, CA, USA). The membranes were incubated at room temperature for 1 h with 5% skimmed milk powder to block non-specific antibody binding. Primary rabbit monoclonal antibody against human AEG-1 (ab124789) (1:10,000; Abcam, Cambridge, MA, USA) or mouse monoclonal antibody against human β - actin (ab6276) (1:5,000; Abcam, Cambridge, MA, USA) and mouse monoclonal antibodies HPV 16-E6 (sc-460), HPV 16-E7 (sc-65711), HPV 18-E6 (sc-365089), HPV 18-E7 (sc-365035) (1:500; Santa Cruz Biotechnology, St Louis, CA) were incubated overnight at 4°C and then incubated for 1 h at room temperature with a goat anti-rabbit (ab6722) or anti-mouse (ab97020) alkaline phosphatase tagged secondary antibody (1:1,000; Abcam, Cambridge, MA, USA). Bands detected using the of BCIP/NBT solution (Merck Millipore, Bedford, MD, USA) were visualized using Bio-Rad Gel doc XR plus (Bio-Rad, Hercules, CA, USA) and band intensities were quantified using ImageJ software (NIH).

### Statistical Analysis

Statistical analyses were performed using GraphPad Prism Software 7.0 (GraphPad Software, La Jolla, CA, USA), and the One-way ANOVA was performed for comparison. *P* < 0.05 was considered Significant. All the data are presented at least three separate experiments.

## Results

### miR-375 Expression Is Markedly Down-Regulated in CC Cell Lines and AEG-1 Is a Direct Downstream Target of miR-375

**Figure 1:**
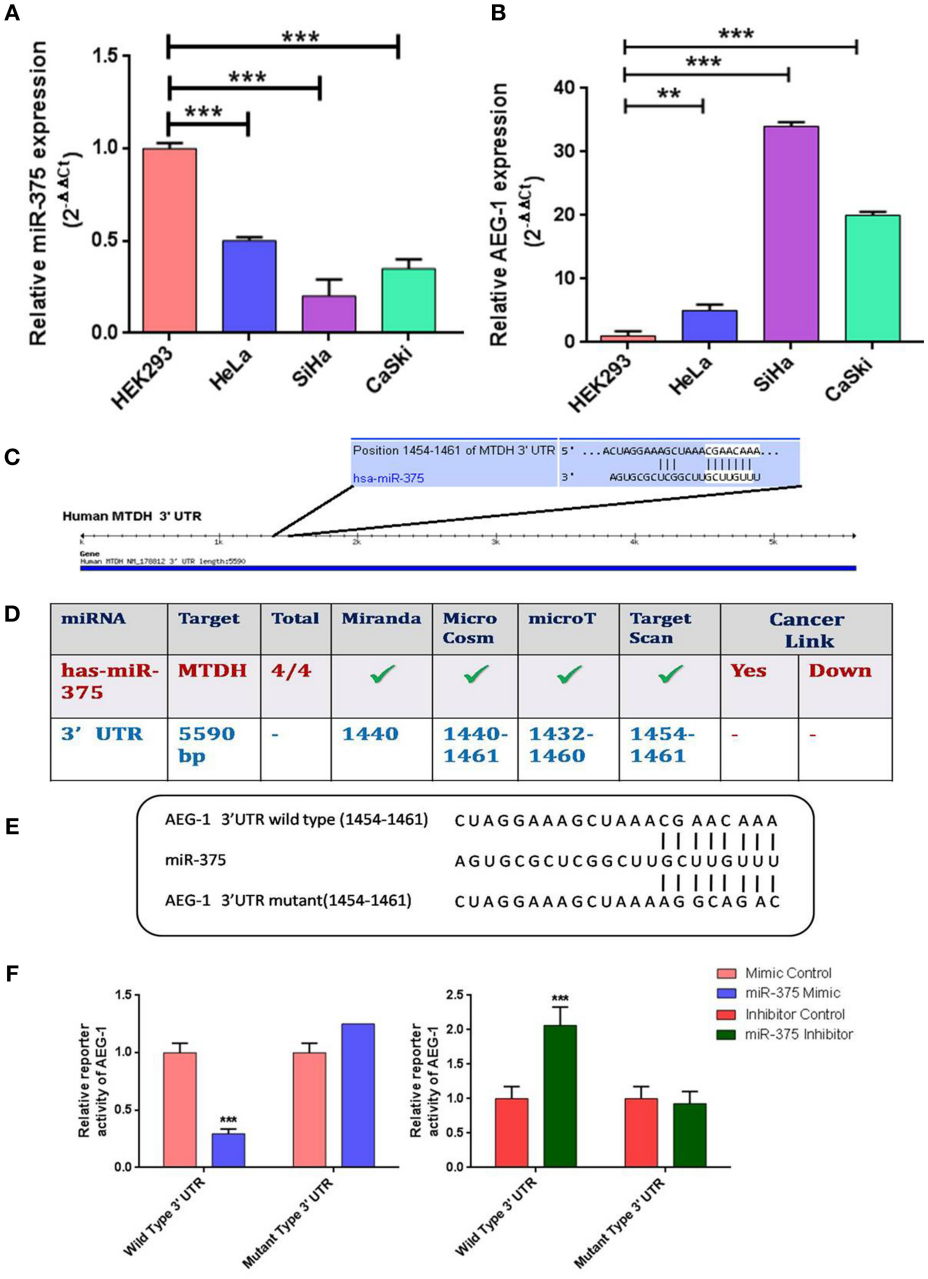
Relative expression levels of miR-375 and AEG-1 in normal and cervical cancer cell lines. Quantitative Real-Time PCR was performed to analyze the expression level of miR-375 **(A)** and AEG-1 **(B)** from normal and cervical cancer cells, RNU6 and GAPDH were used to normalize the expression. The putative AEG-1 3'UTR binding sites of miR-375 **(C)** were confirmed by using different target prediction bioinformatics tools **(D)**. Wild-type (WT) and mutant (MUT) miR-375-binding sites in AEG-1 3′ UTR and miR-375 binding sequence are shown **(E)**. psiCHECK-2 vector containing AEG-1 3'UTR either with wild-type miR-375-binding site (WT) or mutated site (MUT) was co-transfected with miR-375 mimic, miR-375 Inhibitor and their Negative Controls in HEK293T cells and luciferase assay were performed **(F)**. Error bars represent mean ± s.d. and *P-*values are represented as ^**^P< 0.05, ^***^P< 0.001 compared to the corresponding controls.

First, we examined the expression of miR-375 and its target AEG-1 mRNA in four CC cell lines (HeLa, SiHa, CaSki) (Figures 1A,B), C33A (Supplementary Figures 1A,B) and HEK-293, a widely used non-cancer human cell line (34–38). The results showed that the expression of miR-375 was significantly decreased in all CC cell lines when compared to HEK-293. We next examined the molecular mechanisms by which miR-375 regulates CC cellular proliferation, invasion and migration. First, we utilized the Miranda, MicroCosm, microT, TargetScan, and microRNA database to predict the targets of miR-375 and AEG-1 and its binding sites (Figure 1C) was identified as a potential target (Figures 1D,E). Next, we checked whether the AEG-1 was a direct target of miR-375 by using the luciferase reporter assay. The sequence of a target region of the AEG-1 Wild Type 3′-UTR (WT) or a mutant sequence containing miR-375 site (Mutant type) (Figure 1F) was cloned into a luciferase reporter vector. Constructed reporter vector was then co-transfected with miR mimic control and miR-375 mimic or miR inhibitor control and miR-375 inhibitor into the HEK293T cells. The results revealed that miR-375 mimic decreased the luciferase activity of the AEG-1 Wild-type 3′-UTR construct. Mutation of the target site abolished the inhibitory effect of miR-375 mimic on luciferase activity. However, miR-375 inhibitor group increased the luciferase activity of AEG-1 in HEK293T.

### miR-375 Was Upregulated and AEG-1 Was Downregulated by Ectopic Expression of miR-375 Using miR-375 Mimic in CC Cells

**Figure 2:**
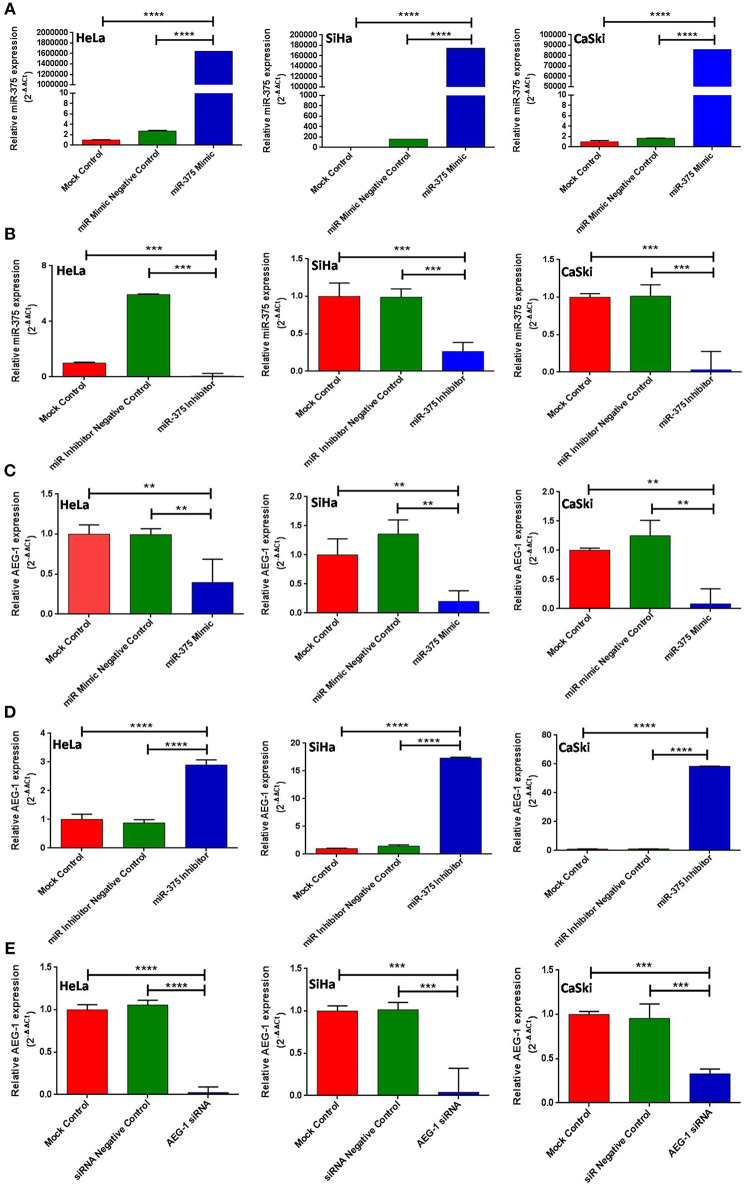
miR-375 regulates AEG-1 expression in cervical cancer cell line. HeLa, SiHa, and CaSki cells were transfected with Mock Control, miR-Mimic Negative Control, miR-Inhibitor Negative Control, miR-375 mimic, miR-375 Inhibitor, siR-Control, and AEG-1 siRNA and the expression level of miR-375 and AEG-1 were estimated by qRT-PCR using RNU6 and GAPDH, respectively, for normalization. miR-375 mimic increased endogenous miR-375 expression in CC cells **(A)** miR-375 Inhibitor decreased endogenous miR-375 expression **(B)**. Ectopic expression of miR-375 decreased AEG-1 mRNA expression **(C)** AEG-1 mRNA expression increased while inhibiting endogenous miR-375 by using miR-375 inhibitor **(D)**. AEG-1 siRNA decreased AEG-1 mRNA expression **(E)** in CC cells. Error bars represent mean ± s.d. and *P-*values are represented as ^**^P< 0.05, ^***^P< 0.001, ^****^P< 0.0001 compared to the corresponding controls.

Next, we confirmed the expression level of miR-375 by establishing transient CC cells (HeLa, SiHa, CaSki) expressing miR-375 by using miR-375 mimic. Similarly, the miR-375 inhibitor has downregulated miR-375 (Figures 2A,B). Subsequently, we analyzed the AEG-1 expression in miR-375 mimic and miR-375 Inhibitor transfected group, AEG-1 was significantly downregulated in the miR-375 transfected group and on the other hand, AEG-1 expression was drastically overexpressed in miR-375 inhibitor treated cells (Figures 2C,D). Overexpression and downregulation of miR-375 and AEG-1 were also confirmed by qRT-PCR. Moreover, we have performed gain-and loss-of-function of miR-375 in C33A cells (Supplementary Figures 2A,B). AEG-1 expression was downregulated and upregulated in miR-375 mimic and inhibitor transfected C33A cells, respectively (Supplementary Figures 2C,D). Also, we confirmed the silencing efficacy of AEG-1 siRNA in CC (HeLa, SiHa, CaSki) (Figure 2E) and C33A cells (Supplementary Figure 2E) for further experiments.

### miR-375 Regulated the AEG-1 Mediated Post-Wound Migration, Invasion, and Angiogenesis *in vitro*

**Figure 3:**
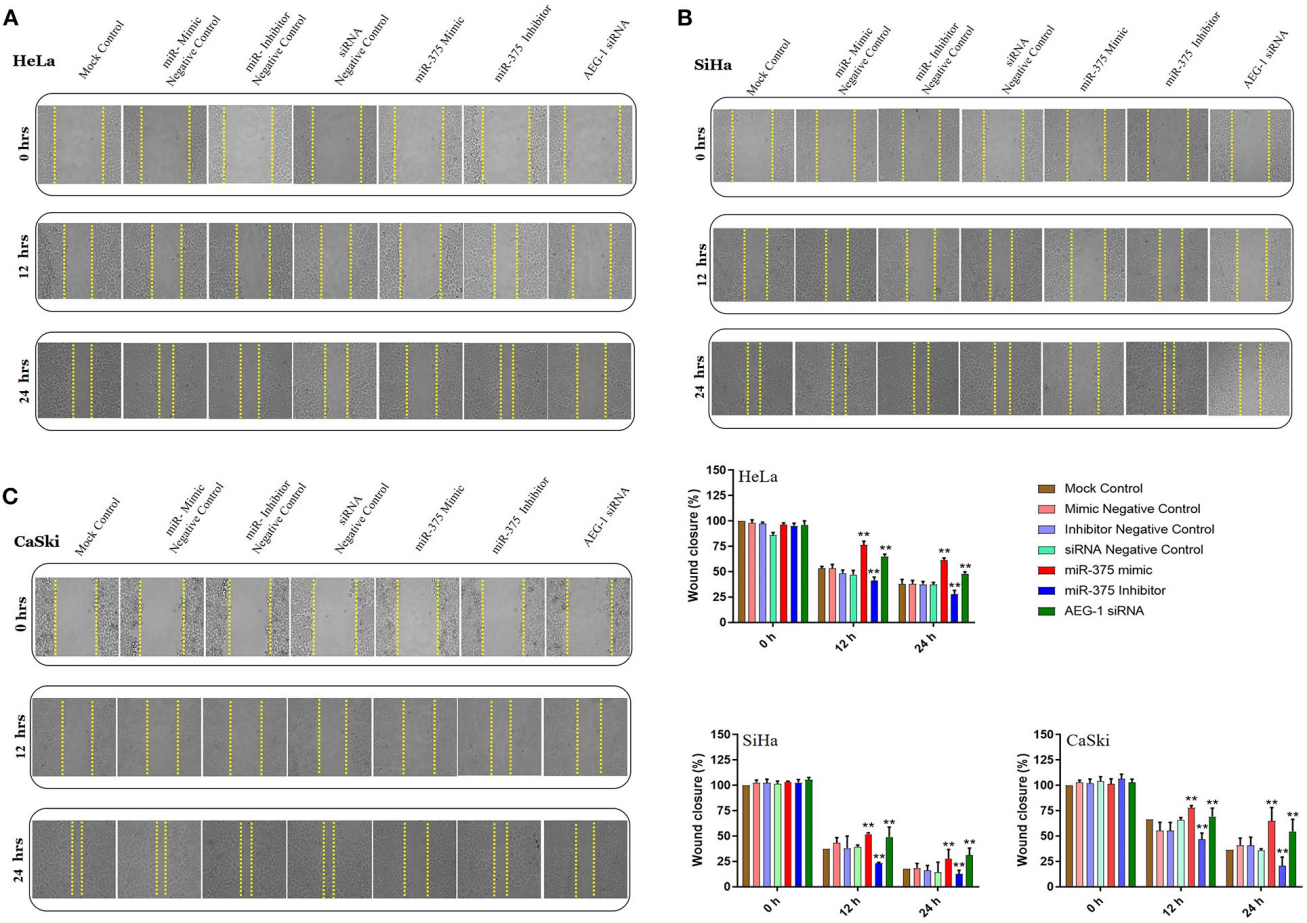
Ectopic expression of miR-375 inhibits cervical cancer cell migration. *in vitro* scratch assay with HeLa **(A)**, SiHa **(B)** and CaSki **(C)** cell lines at 0, 12, and 24 h post-transfection with miR-375 mimic, miR-375 Inhibitor, AEG-1 siRNA and their controls. Gap distance of cells was quantified by using Image J. The scale bars represent 100 μ*m*. Error bars represent mean ± s.d. and *P-*values are represented as ^**^P< 0.05 compared to the corresponding controls at a different time interval.

**Figure 4:**
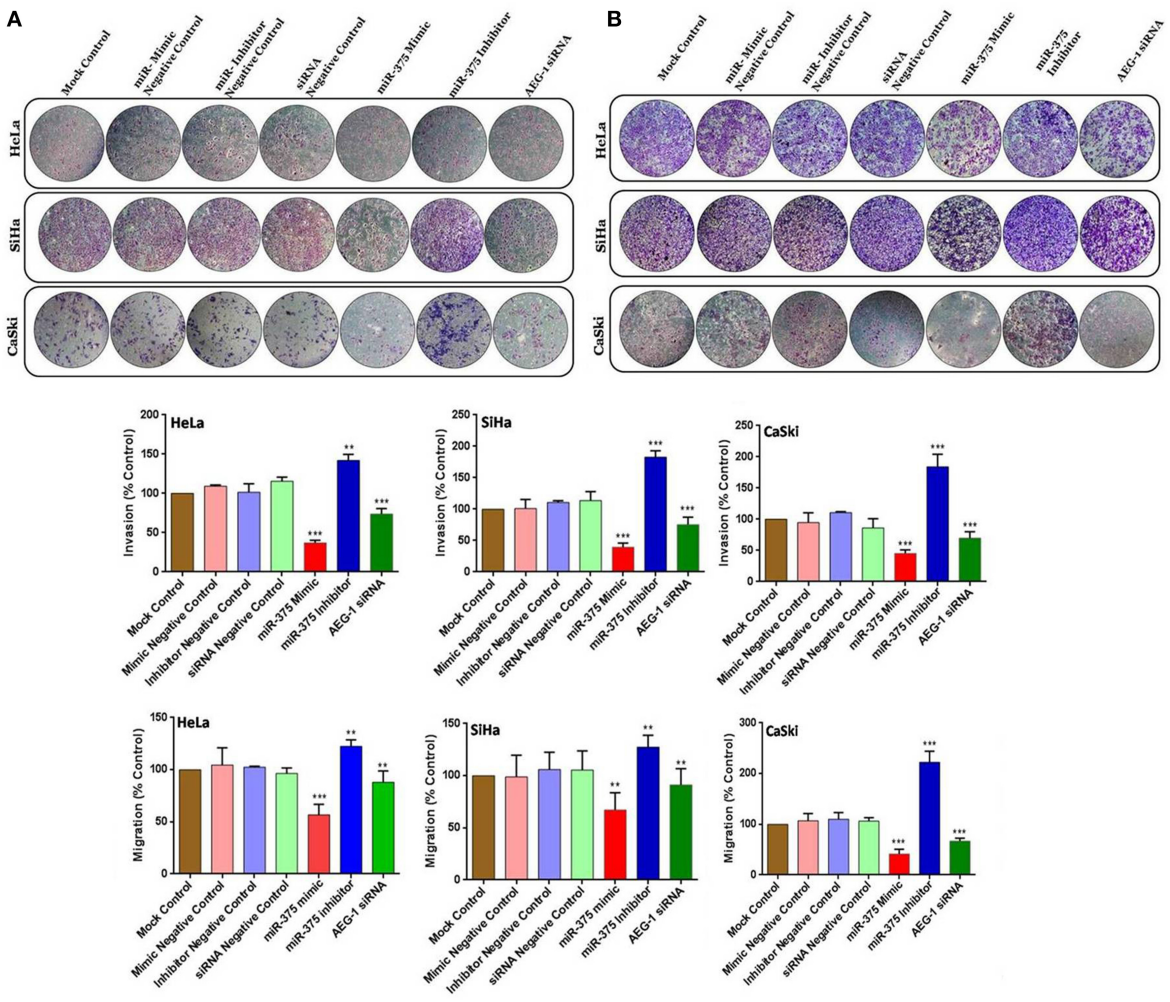
miR-375 suppressed the invasion and migration in cervical cancer cells. Transwell invasion assay with Matrigel was performed in miR-375 mimic, miR-375 Inhibitor, AEG-1 siRNA and their controls transfected CC cells **(A)**. Transwell migration assay without Matrigel was performed in miR-375 mimic, miR-375 Inhibitor, AEG-1 siRNA, and their controls transfected CC cells **(B)**. The scale bars represent 100 μ*m*. Error bars represent mean ± s.d. and *P-*values are represented as ^**^P< 0.05, ^***^P< 0.001 compared to the corresponding controls.

Overexpression of AEG-1 is an important process that occurs during CC cells migration, invasion, and angiogenesis (20). To evaluate the roles of miR-375 in cell migration, invasion, and angiogenesis, wound-healing, transwell invasion, migration, and tube formation assay were performed in CC cells. We first examined wound-healing assay, and the result showed that the miR-375 mimics markedly inhibited HeLa (Figure 3A), SiHa (Figure 3B), CaSki (Figure 3C), and C33A cells (Supplementary Figure 3A) migration. Subsequently, the miR-375 inhibitor significantly increased CC cells migration. We next observed the invasiveness and migration of CC (HeLa, SiHa, CaSki) (Figures 4A,B) and C33A (Supplementary Figures 3B,C) cells; transwell invasion and migration assay indicated that the invasiveness and migration ability of CC cells transfected with miR-375 mimic was significantly decreased, whereas with miR-375 inhibitor treatment we found an increased invasion and migration in CC cells.

Finally, we assessed the role of miR-375 in tumor angiogenesis by tube formation assay; we ectopically expressed miR-375 in HUVEC cells. Our results showed that miR-375 expression in HUVEC cells decreased tube formation (as measured by total tube length and branch points) when compared to the control. Inhibiting miR-375 significantly enhances tube formation of HUVECs (Supplementary Figure 4). To validate the effect of AEG-1 in CC cells invasion, migration and tube formation, AEG-1 siRNA 2 also be used. AEG-1 siRNA 2 significantly inhibits CC (HeLa, SiHa, CaSki) (Supplementary Figures 5A–C) and C33A cells (Supplementary Figures 3D,E) invasion, migration and inhibits HUVECs tube formation. This result indicated that miR-375 overexpression inhibited HUVECs angiogenesis by inhibiting AEG-1 expression. Therefore, miR-375 was identified as the target miRNA due to its obvious potential to be highly associated with angiogenesis.

### miR-375 Inhibits Cellular Proliferation and Enhance the Chemosensitivity of CC Cells to 5-Fluorouracil

**Figure 5:**
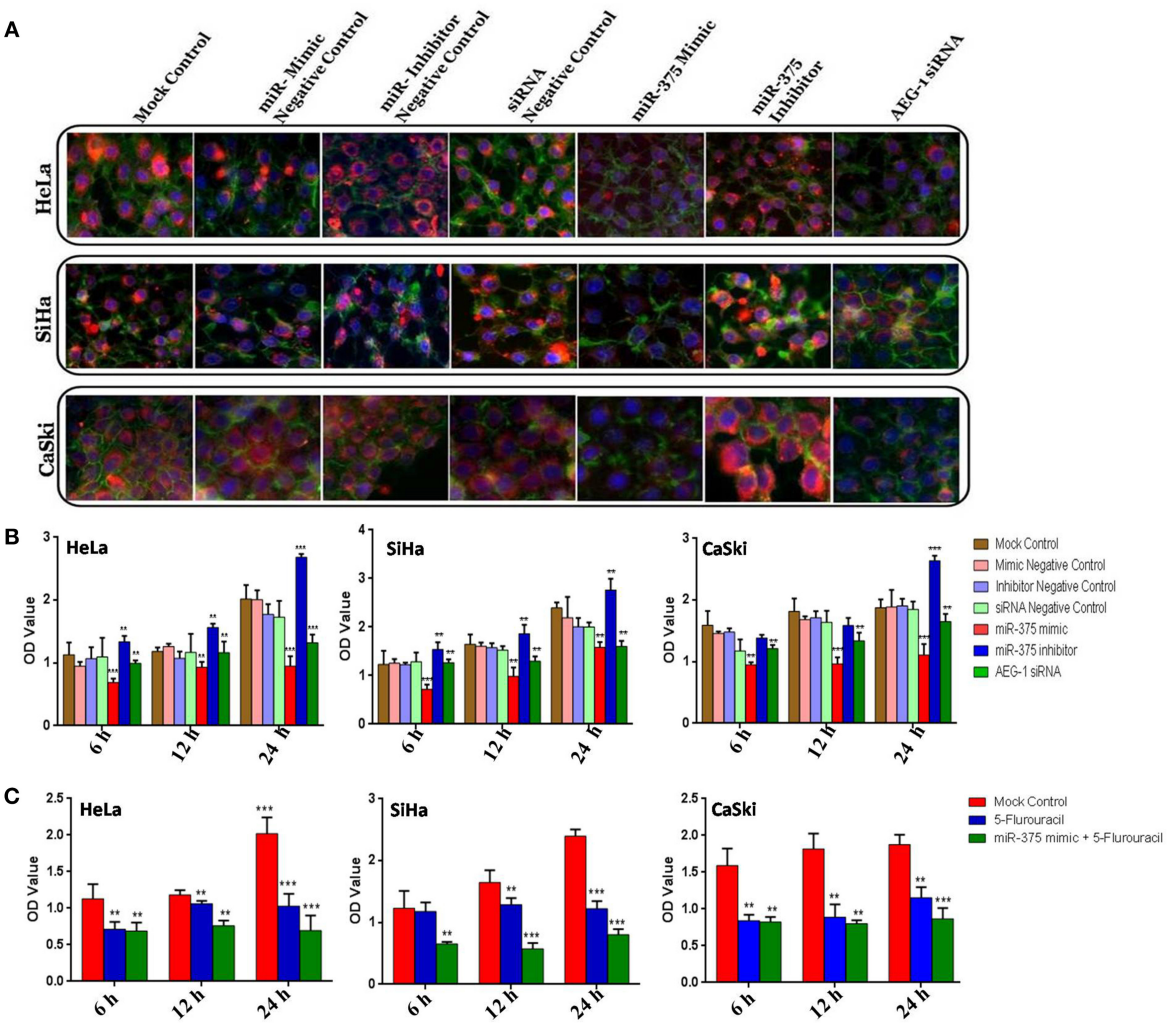
miR-375 inhibits cellular proliferation, enhance 5-Fluorouracil sensitivity and Immunocytochemistry in CC cells. AEG-1 Protein expression was determined by Immunocytochemistry **(A)**. Effect of miR-375 mimic on CC cells proliferation of by MTT assay **(B)**. CC cells were transfected with a mock control, miR-375 mimic and then the miR-375 transfected cells were treated with IC_50_ Value of 5-Fluorouracil and incubated at different time intervals (6, 12, and 24 h) **(C)**. The scale bars represent 100 μ*m*. Error bars represent mean ± s.d. and *P-*values are represented as ^**^P< 0.05, ^***^P< 0.001 compared to the corresponding controls.

We examined the protein expression of AEG-1 in CC (HeLa, SiHa, CaSki) (Figure 5A) and C33A cells (Supplementary Figure 6A) using Immunocytochemistry, and phalloidin was used to stain the actin filaments. The intensity of AEG-1 protein stain was reduced in miR-375 mimic treated cells. In contrast, the intensity of AEG-1 protein stain was increased in miR-375 inhibitor treated cells. Cell proliferation was significantly lowered in the cells transfected with the miR-375 mimic when compared to mock and mimic control. Consistently, when the miR-375 inhibitor was transfected into CC (HeLa, SiHa, CaSki) (Figure 5B) and C33A (Supplementary Figure 6B) cells, the proliferation ratio was higher than that the mock and inhibitor control group. Furthermore, miR-375 enhances 5-fluorouracil sensitivity of CC (HeLa, SiHa, CaSki) (Figure 5C) and C33A cells (Supplementary Figure 6C) and was confirmed by the MTT assay. The MTT chemosensitivity assay indicated that the IC_50_ values of 5-Fluorouracil decreased significantly due to transfection with the miR-375 mimic.

### Ectopic Expression of miR-375 Induces Cell Cycle Arrest and Apoptosis in CC Cells

**Figure 6:**
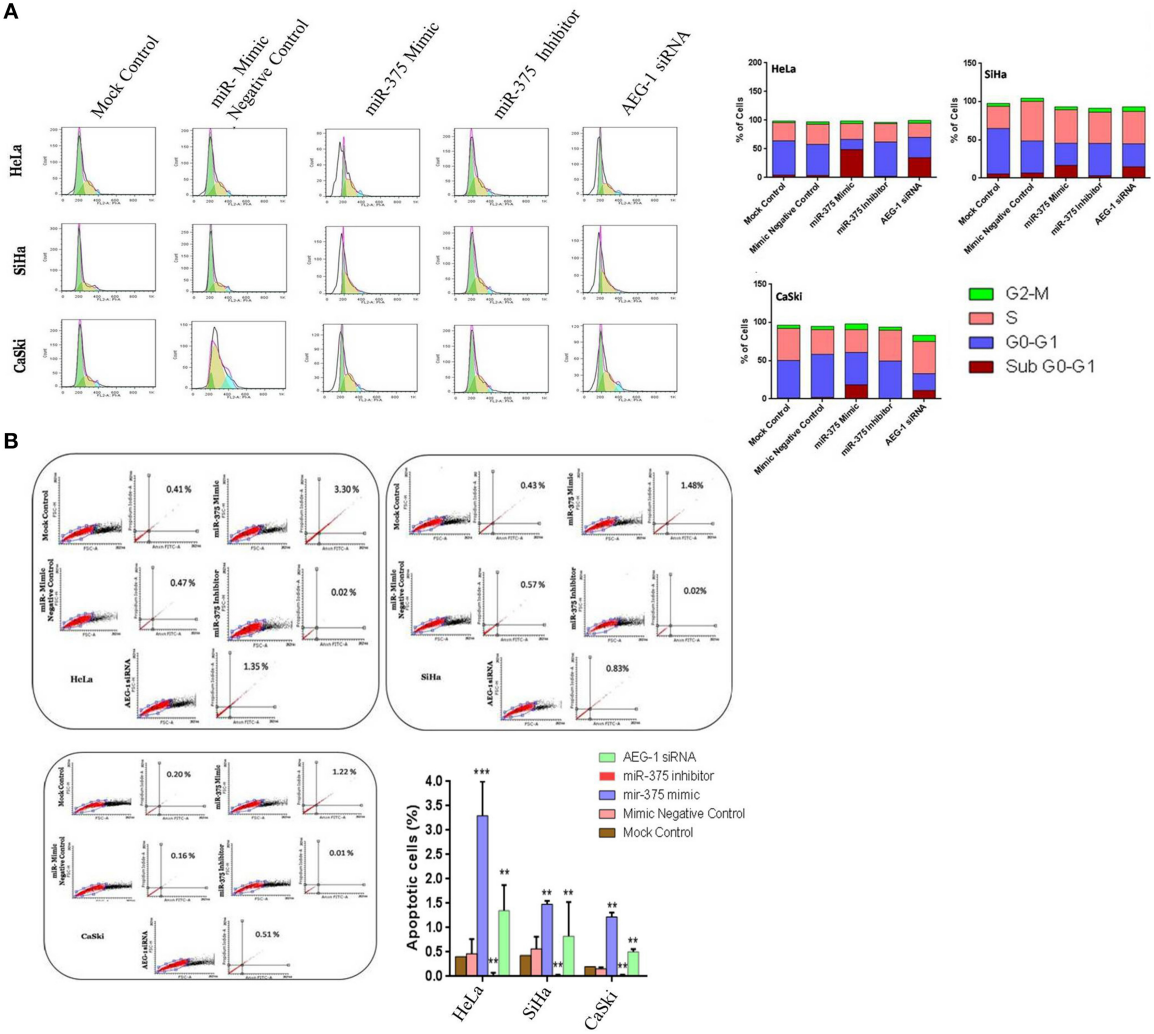
miR-375 induces cell cycle arrest and apoptosis *in vitro*. CC cells were transfected with miR-375 mimic, miR-375 Inhibitor, AEG-1 siRNA, and their controls and 48 h later, the effect of miR-375 on cell cycle was evaluated by the Flow cytometry **(A)**. Apoptosis was assessed in cells transfected with miR-375 mimic, miR-375 Inhibitor, AEG-1 siRNA, and their controls by Flow cytometry **(B)**. Error bars represent mean ± s.d. and *P-*values are represented as ^**^P< 0.05, ^***^P< 0.001 compared to the corresponding controls.

To illustrate the function of miR-375 in cell cycle and apoptosis by using FCM analysis, miR-375 was overexpressed in CC (HeLa, SiHa, CaSki) (Figure 6A) and C33A cells (Supplementary Figure 7A) by transfecting miR-375 mimic. miR-375 mimic transfected CC cells showed a higher sub-G1 phase, which means miR-375 induces cell cycle arrest in sub-G1 phase, while the sub-G1 phase is lowering in miR-375 inhibitor transfected group. Furthermore, apoptosis was analyzed by using Alexa Fluor 488-conjugated Annexin V/PI dual staining apoptosis kit; we confirmed that ectopic expression of miR-375 induces apoptosis in CC cells. On the contrary, inhibition of miR-375 significantly reduce CC cells apoptosis; these pieces of evidence indicated that re-expression of miR-375 induces apoptosis in CC (HeLa, SiHa, CaSki) (Figure 6B) and C33A cells (Supplementary Figure 7B).

### miR-375 Alters AEG-1 Protein Expression and HPV Viral Oncogenes E6 and E7 Inhibit the Endogenous Expression of miR-375 in CC Cells

**Figure 7:**
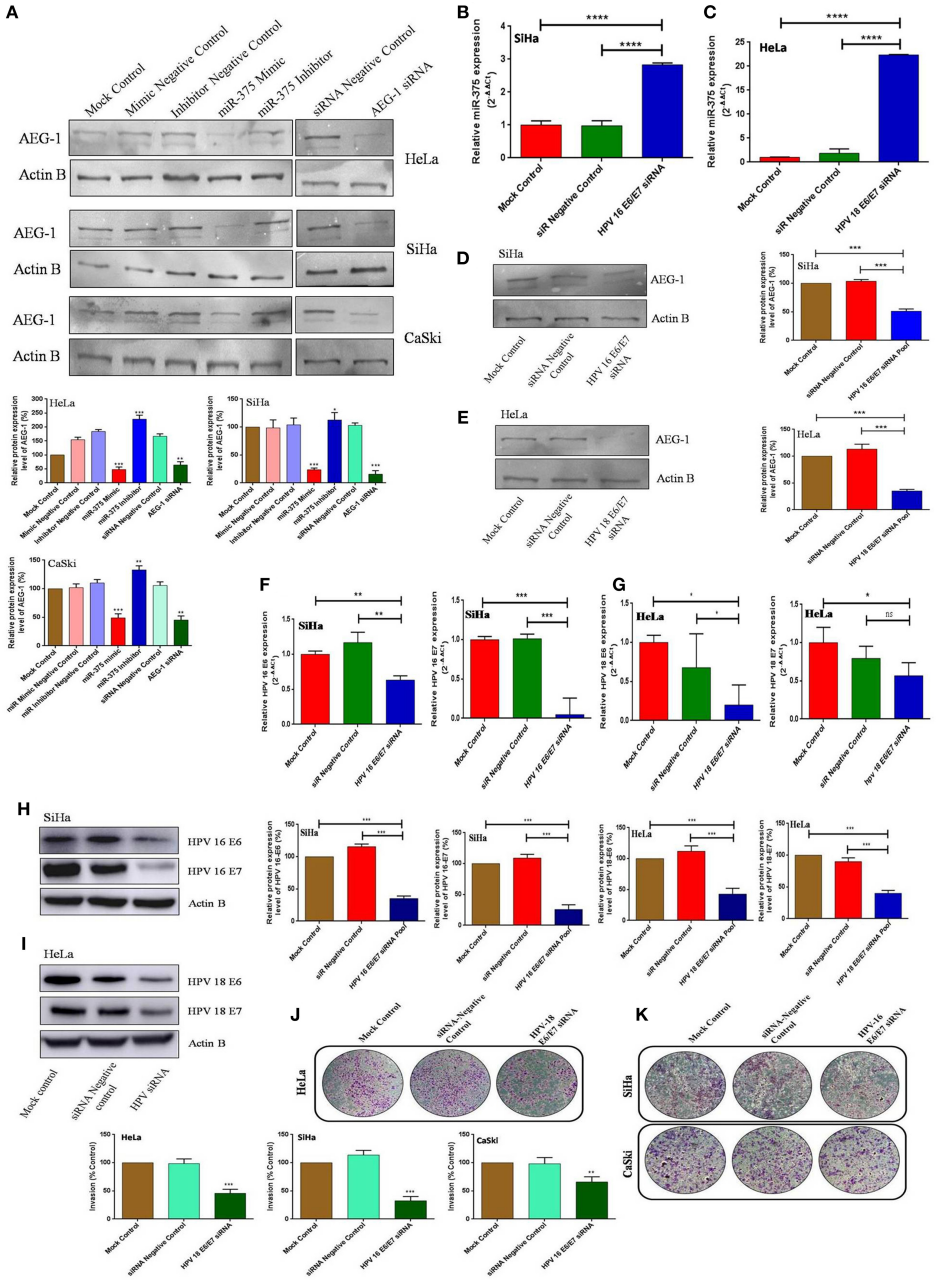
miR-375 suppresses AEG-1 protein level and HPV regulates the miR-375 expression. Western blot validation of AEG-1 protein expression in CC cells after transfecting miR-375 mimic, miR-375 Inhibitor, AEG-1 siRNA and their controls with β-actin as the loading control **(A)**. Effect of HPV16, 18-E6/E7 siRNA on miR-375 expression in SiHa **(B)** and HeLa **(C)** cells were determined by qRT-PCR. Effect of HPV16, 18-E6/E7 siRNA on miR-375 targets AEG-1 protein expression in SiHa **(D)** and HeLa **(E)** cells were determined by western blot. Effect of HPV16, 18-E6/E7 siRNA on E6 and E7 mRNA expression in SiHa **(F)** and HeLa **(G)** cells were determined by qRT-PCR and protein expression were determined by western blot **(H,I)**. Transwell invasion assay with Matrigel was performed in HPV 18 E6/E7 siRNA, HPV 16 E6/E7 siRNA and their controls transfected CC cells **(J,K)**. Error bars represent mean ± s.d. and *P-*values are represented as ^*^p< 0.05, ^**^P < 0.05, ^***^P < 0.001 and ^****^p < 0.0001, ns – non significant compared to the corresponding controls.

The ectopic expression of miR-375 and inhibition of AEG-1 that decreased the AEG-1 protein expression in CC (HeLa, SiHa, CaSki) (Figure 7A) and C33A cells (Supplementary Figure 8). However, the AEG-1 protein expression level increased while inhibiting miR-375 by using a miR-375 inhibitor. To validate the association between HPV and miR-375, we performed qRT-PCR analysis and the results indicated that the inhibition of HPV 16-E6 and E7 significantly increased the endogenous expression of miR-375 in SiHa cells (Figure 7B). Interestingly the same results occurred in HeLa cells inhibition of HPV18-E6 and E7 significantly increased the miR-375 expression (Figure 7C). We confirmed that the expression of miR-375 target; AEG-1 protein were decreased upon inhibition of HPV 16/18 E6/E7 in SiHa and HeLa cells by using western blot (Figures 7D,E) We further confirmed the inhibition of HPV 16 and 18 E6-E7 by using corresponding siRNAs in qRT-PCR and western blot in SiHa, HeLa (Figures 7F–I) and CaSki cell line (Supplementary Figure 9). Furthermore, HPV 16, 18 E6/E7 siRNA significantly inhibits invasion of CC cells (Figures 7J,K). We also confirmed the p53 restoration and Rb expression level in our previous studies by using the HPV 16/18 E6-E7 siRNA (39, 41). We hypothesized that HPV 16 and 18 E6-E7 viral oncogenes could induce differential miR-375 expression which contributed to the cervical tumorigenesis.

## Discussion

Accumulating evidence has demonstrated that miRNAs act as oncogenes or tumor suppressors by targeting genes involved in proliferation, migration, chemoresistance, survival, apoptosis, metastasis, and cell differentiation (42, 43), suggesting a new mechanism involved in the initiation and development of CC. Previous studies have reported the down-regulation of miR-375 in various human cancers. This prompted us to determine the expression of miR-375 in CC.

miR-375 was first identified from murine pancreatic β-cell line MIN6. It acts as a pancreatic islet-specific miRNA. Further studies confirmed that miR-375 is widely present in various tissues or organs and that its expression was significantly downregulated in various cancers such as gastric cancer, glioma, melanoma, and esophageal carcinoma (15, 44–46). This evidence clearly shows that miR-375 is an important cancer-related miRNA and that it has vital roles in regulating CC development and progression. In this study, we found that miR-375 was significantly downregulated in CC cells, which indicated its potential antitumor function.

miR-375 plays an important role in carcinogenesis. Our gain-and loss-of-function experiments demonstrated that miR-375 mimics significantly inhibited CC cell proliferation, invasion, and migration, while miR-375 inhibition significantly augmented cell proliferation, invasion, and migration. By using several bioinformatics tools, we identified AEG-1 as a potential target of miR-375. Furthermore, the overexpression of AEG-1 has been identified in numerous human cancers. It has been reported that AEG-1 contributes to several hallmarks of metastatic cancers, including cell proliferation, survival under chemotherapy, increased migration, and invasiveness (19). Our luciferase assay demonstrated that the downregulation of AEG-1 was mediated by miR-375. In the present work, we have identified that AEG-1 expression was upregulated in CC cells and that it could promote cell proliferation, migration, and invasion. AEG-1 gene expression was also significantly downregulated in miR-375 mimic transfected CC cells, whereas AEG-1 expression was increased in miR-375 inhibitor treated cells. Moreover, we observed an inverse correlation between miR-375 and AEG-1 expression in CC. Our study is the first to have explored AEG-1 as a potential target of miR-375, suggesting a pivotal role of AEG-1 in cervical tumorigenesis. In line with our study, downregulation of AEG-1 by miR-375 in hepatocellular carcinoma, breast cancer, head and neck squamous cell carcinoma, esophageal cancer and adrenocortical carcinoma led to inhibition of cancer cell growth, proliferation and invasiveness (29, 30, 47–49). Thus, we concluded that miR-375 inhibits cellular proliferation, migration, and invasion by targeting the 3′- UTR of AEG-1.

Alam et al. (50) reported that ectopic expression of miR-375 targets connective tissue growth factor (CTGF) and significantly inhibits cell proliferation in colon cancer. In colon cancer cells (HCT116 and HT29), miR-375 was significantly downregulated and inhibited angiogenesis. Furthermore, flow cytometric analysis showed significant increase in apoptosis in miR-375 transfected cells. Moreover, the overexpression of miR-375 arrested cell cycle in the G1 phase in colon cancer cells (50). Similar results were obtained from our study, while miR-375 mimic or AEG-1 siRNA transfected CC cells inhibited cell proliferation, angiogenesis, induced apoptosis and sub G0-G1 phase arrested in CC cells, miR-375 inhibition reversed these effects.

Chemoresistance is a considerable problem in cancer therapeutic strategy and it remains one of the key factors in cancer death. The majority of tumors show good response to chemotherapy initially; but after continued treatment they start to develop resistance (51, 52). In this study, we explored whether miR-375 enhances the chemosensitivity of 5-fluorouracil in CC. Previous studies have demonstrated that miR-375 is involved in the development of chemoresistance in cancers. Shen et al. (53) found that paclitaxel treated cervical cells and tissues showed high expression of miR-375. Overexpression of miR-375 in CC cells decreased the sensitivity toward paclitaxel *in vitro* and *in vivo*. Paclitaxel upregulated miR-375 expression and overexpression of miR-375 increased chemoresistance in CC (53). Wang et al. (43) showed that miR-375 induces docetaxel resistance by targeting SEC23A and YAP1 in prostate cancer. miR-375 overexpression significantly reduced prostate cancer cell sensitivity to docetaxel treatment (54). In contrast, miR-375 enhanced the chemosensitivity to platinum-based cisplatin. Overexpression of miR-375 targets ERBB2 and enhances cisplatin sensitivity in human gastric cells (55). miR-375 and doxorubicin co-delivered using liposomes increased the sensitivity of doxorubicin by decreasing the expression of multidrug resistance gene 1 (MDR1) by targeting AEG-1 in hepatocellular carcinoma (56). Our results also show that the ectopic expression of miR-375 enhances 5-fluorouracil chemosensitivity in CC cells by targeting AEG-1. Further studies are required to explore the crosstalk between miR-375 and chemotherapeutic drugs to find out the exact molecular mechanisms involved in CC chemoresistance.

We also identified the interplay between HPV 16/18-E6/E7 and miR-375 in CC. Only a few studies have evaluated the potential effects of HPV viral oncogenes on miR-375 dysregulation. Liu et al. (57) reported that HPV 16 E6 modulates the expression of DNMT1 in SiHa and CaSki cells. Inhibition of DNMT1 partially restored miR-375 in the cells. HPV-16 enhanced DNMT1 upregulation which in turn triggered the downregulation of miR-375 through promoter hypermethylation in CC cells (57). Jung et al. (58) discovered that replenishment of miR-375 in HPV-positive cancer cell lines (SiHa and HeLa) and oropharyngeal cell lines significantly reduces the levels of HPV 16 and 18 transcripts. Furthermore, the overexpression of miR-375 by using miR-375 mimics in HPV-positive cancer cells induces the regulation of HPV E6/E7, which protected the expression of tumor suppressor p53 and RB. Accordingly, downregulation of miR-375 during HPV-mediated carcinogenesis is likely to trigger the dysregulation of this system (58). Another study showed the influence of HPV genes on different miRNA expressions. For example, HPV 16 deregulates miR-139-3p in HPV-associated cancers (59), HPV 16 E5 down-regulates miR-196a expression and up-regulated HoxB8, a target of miR-196a in cervical cancer (60). Interestingly, in our study, we found that miR-375 expression significantly increased in HPV 16/18-E6/E7 silenced SiHa, CaSki, and HeLa cells. Moreover, inhibition of HPV oncogenes E6 and E7 by HPV 16, 18 E6/E7 siRNAs in CC cells significantly inhibited cell invasion. Taken together, HPV 16 and 18 viral oncogenes, E6 and E7, inhibit endogenous miR-375 expression in CC. Here, we report that miR-375 expression is lower in HPV 16/18 positive cell lines compared to the HPV negative and normal cell lines. However, the expression of miR-375 was aberrantly decreased in HPV 16 positive cell lines SiHa and CaSki, compared to HPV 18 positive cell line, HeLa. Moreover, our results showed the miR-375 expression in the HPV 16/18 groups was meagerly low in HPV 18 positive cell lines and drastically low in HPV 16 positive cell lines compared to an HPV negative cell line, C33A. Downregulation of miR-375 in CC is induced by the HPV oncogenes and miR-375 expression may be suppressed by HPV infection. Nevertheless, further studies are needed to determine the exact molecular mechanism underlying the decreased miR-375 expression during HPV infection. We also performed miR-375 ectopic expression, AEG-1 silencing, invasion, migration, cell cycle, and apoptosis analysis in HPV negative C33A cell line (Supplementary Figures 2–3, 6–8). According to previous studies, both miR-375 and AEG-1 are involved in regulating multiple cancers. However, whether there is an association between miR-375 and AEG-1 in CC is not yet known. We identified that there is a negative feedback regulation in CC cells, which constitutes a part of the network among HPV16/18-E6/E7, miR-375, and AEG-1 axis.

## Conclusion

We revealed that miR-375 expression is downregulated in CC cells and that it is correlated with tumorigenesis. miR-375 suppressed cell proliferation, migration, invasion, and angiogenesis; enhanced chemosensitivity toward 5-fluorouracil, arrested cell cycle in sub G0-G1 phase, and induced apoptosis by targeting AEG-1. Our study reported that HPV 16/18-E6/E7 could target downregulate miR-375 expression enhancing AEG-1 oncogene expression and thus leading to tumorigenesis in cervical cells.

Our study revealed mechanistically for the first time how HPV 16/18-E6/E7/miR-375/AEG-1 axis is involved in CC. Taken together, these findings suggest that miR-375 plays a major tumor suppressor role and is a promising potential therapeutic target for CC. Therefore, miR-375 represents a novel therapeutic strategy in the treatment of cervical cancer. However, the detailed direct mechanism of HPV 16/18-E6/E7 and miR-375 remains unknown. Therefore, further investigations are needed to understand the relationship between HPV 16/18-E6/E7 and miR-375 regulation. In our future study, we will further explore the HPV 16/18-E6/E7/miR-375/AEG-1 axis in CC.

## Data Availability

The raw data supporting the conclusions of this manuscript will be made available by the authors, without undue reservation, to any qualified researcher.

## Author Contributions

AA and SJ designed the study, investigated, and acquired funding. SJ, MK, and RM carried out formal analysis, validation of data, and software analysis. NR, HJ, YS, and AA helped to collected materials. AA supervised the experiments and project administration. AA, SJ, and NR wrote the manuscript. All authors read and approved the final manuscript.

### Conflict of Interest Statement

The authors declare that the research was conducted in the absence of any commercial or financial relationships that could be construed as a potential conflict of interest.
