# Corrigendum: Dysregulation of miR-375/AEG-1 Axis by Human Papillomavirus 16/18-E6/E7 Promotes Cellular Proliferation, Migration, and Invasion in Cervical Cancer

**DOI:** 10.3389/fonc.2021.694442

**Published:** 2021-05-12

**Authors:** Sridharan Jayamohan, Maheshkumar Kannan, Rajesh Kannan Moorthy, Nirmal Rajasekaran, Hun Soon Jung, Young Kee Shin, Antony Joseph Velanganni Arockiam

**Affiliations:** ^1^ Molecular Oncology Laboratory, Department of Biochemistry, School of Life Sciences, Bharathidasan University, Tiruchirappalli, India; ^2^ Department of Molecular Medicine and Biopharmaceutical Sciences, Graduate School of Convergence Science and Technology, Seoul National University, Seoul, South Korea; ^3^ Laboratory of Molecular Pathology and Cancer Genomics, College of Pharmacy, Seoul National University, Seoul, South Korea; ^4^ Enhancedbio Inc., Seongdong-gu, South Korea

**Keywords:** human papillomavirus, miR-375, astrocyte elevated gene-1, cervical cancer, cell proliferation

In the original article, there was a mistake in **Figure 3** as published. While image processing, we have grouped few pictures inadvertently in **Figure 3**. The corrected **Figure 3** appears below.

The authors apologize for this error and state that this does not change the scientific conclusions of the article in any way. The original article has been updated.

**Figure 3:**
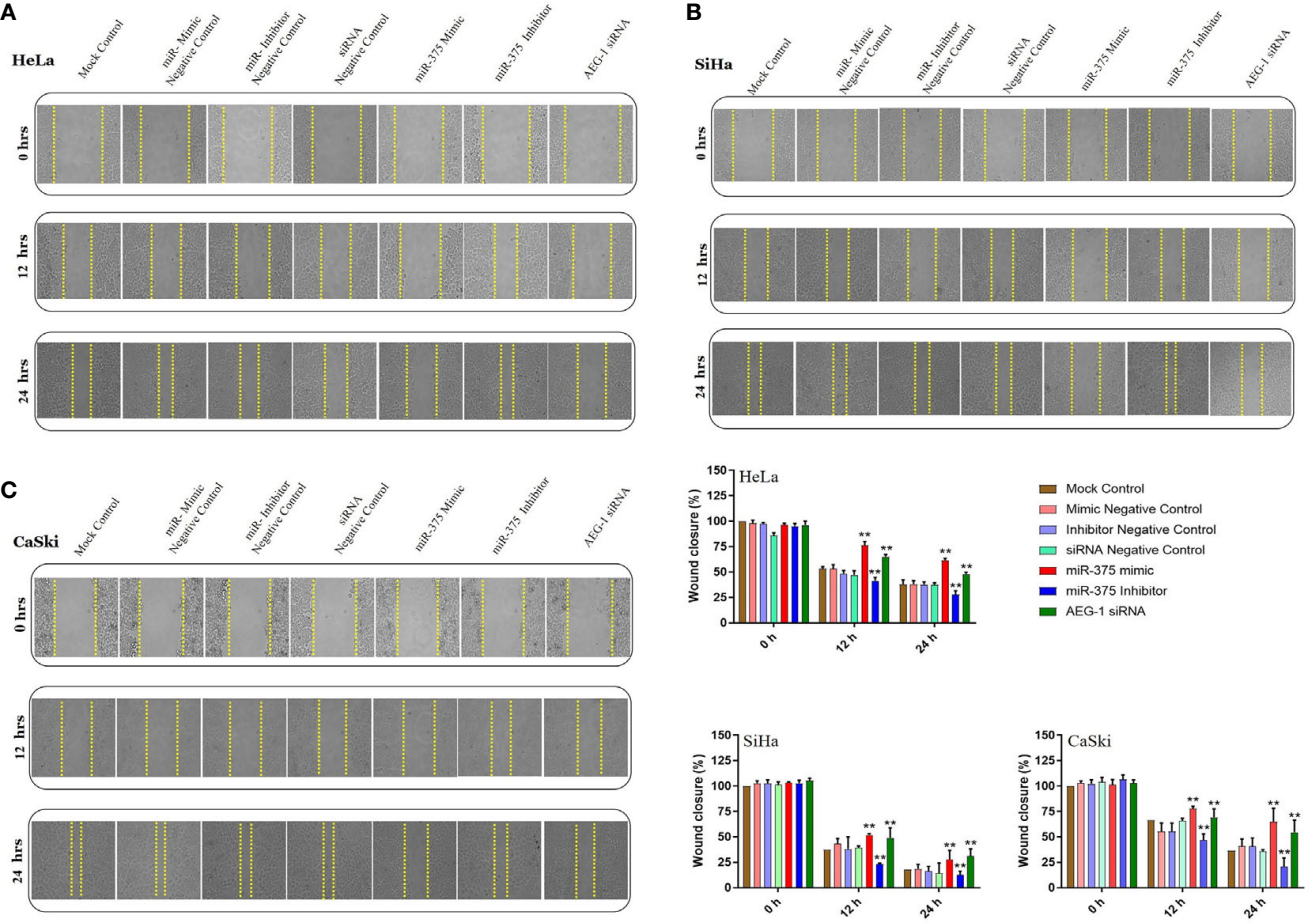
Ectopic expression of miR-375 inhibits cervical cancer cell migration. in vitro scratch assay with HeLa **(A)**, SiHa **(B)** and CaSki **(C)** cell lines at 0, 12, and 24 h post-transfection with miR-375 mimic, miR-375 Inhibitor, AEG-1 siRNA and their controls. Gap distance of celss was quantified by using Image J. The scale bars represent 100 mm. Error bars represent mean ± s.d. and P-values are represented as **P < 0.05 compared to the corresponding controls at a different time interval.

