# Supplementary material for: Dysregulation of miR-375/AEG-1 Axis by Human Papillomavirus 16/18-E6/E7 Promotes Cellular Proliferation, Migration, and Invasion in Cervical Cancer
Source: Front Oncol. 2019 Sep 9;9:847. doi: 10.3389/fonc.2019.00847 (PMC6746205; doi:10.3389/fonc.2019.00847)
Supplement: Supplementary file 1 [file Data_Sheet_1.docx]

**Supplementary Materials**

**Supplementary Figure. 1**


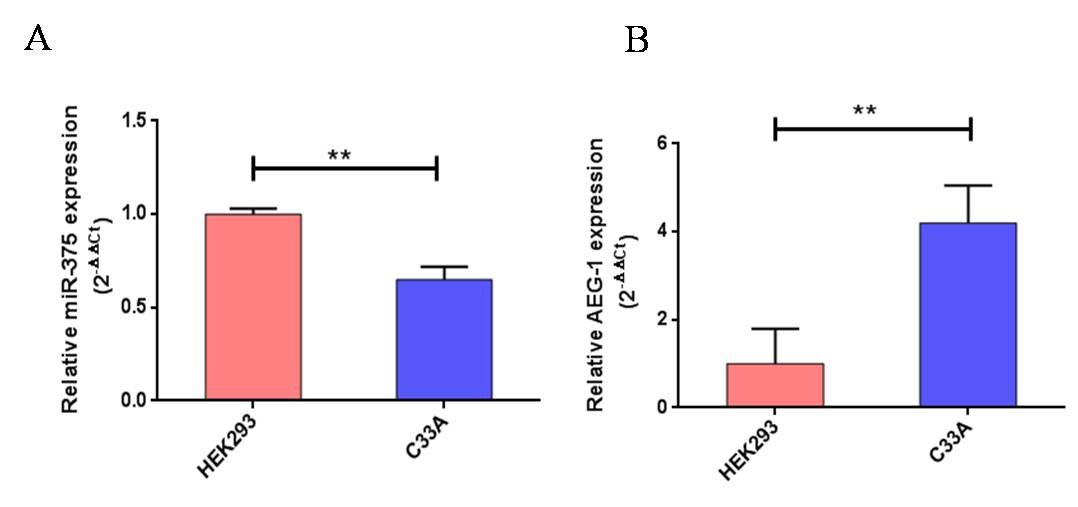


**S. Fig1. Relative expression levels of miR-375 and AEG-1 in normal and C33A.** Quantitative Real-Time PCR was performed to analyze the expression level of miR-375 **(A)** and AEG-1 **(B)** from normal and C33A cells, RNU6 and GAPDH were used to normalize the expression. Error bars represent mean + s.d. and *P-*Values are represented as ***P<*0.05, compared to the corresponding controls

**Supplementary Figure. 2
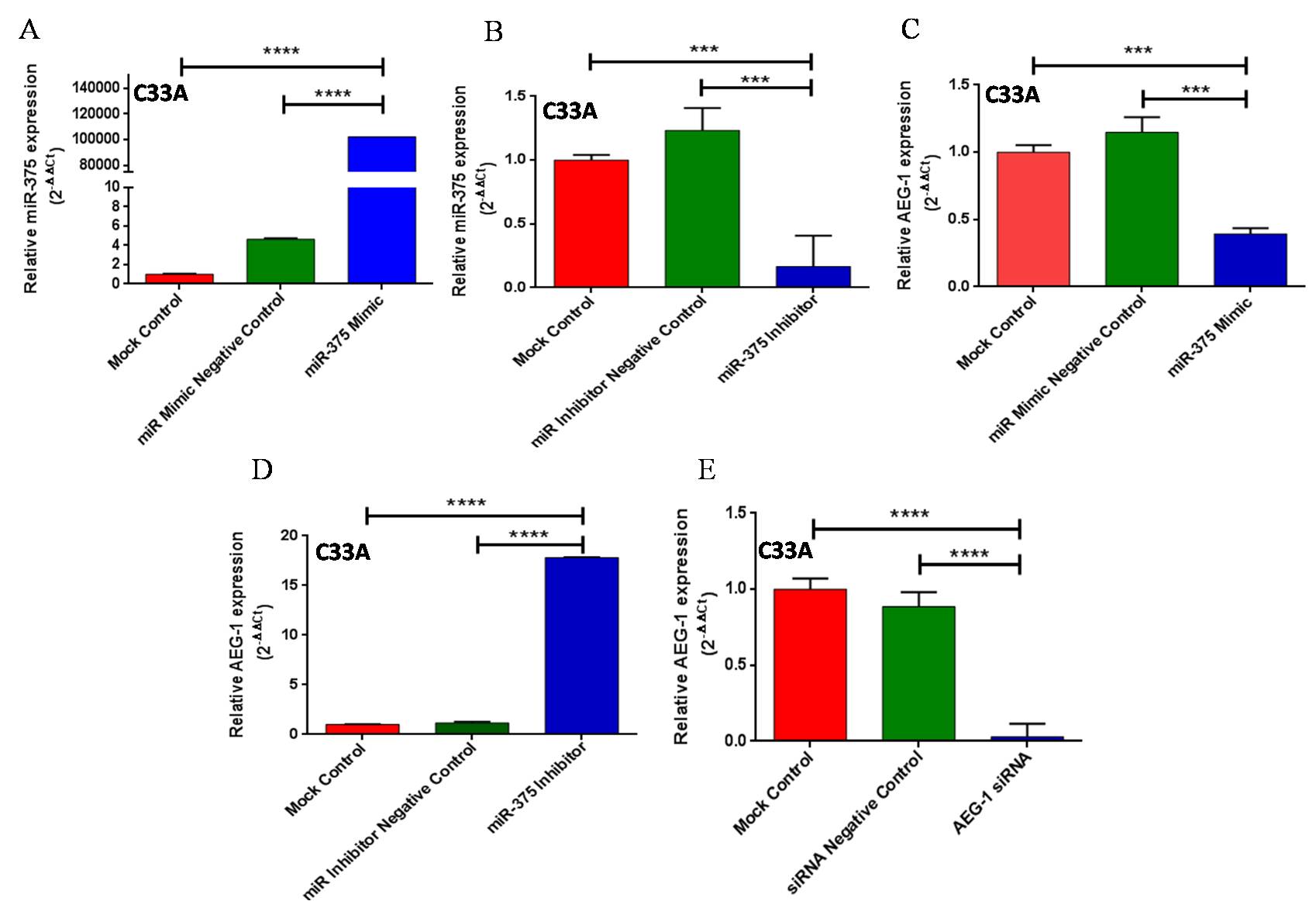
**

**S. Fig 2. miR-375 regulates AEG-1 expression in C33A.** C33A cells were transfected with Mock Control, miR-Mimic Negative Control, miR-Inhibitor Negative Control, miR-375 mimic, miR-375 Inhibitor, siRNA Negative Control and AEG-1 siRNA and the expression level of miR-375 and AEG-1 were estimated by qRT-PCR using RNU6 and GAPDH, respectively for normalization. miR-375 mimic increased endogenous miR-375 expression in C33A cells (**A**) miR-375 Inhibitor decreased endogenous miR-375 expression (**B**). Ectopic expression of miR-375 decreased AEG-1 mRNA expression (**C**) AEG-1 mRNA expression increased while inhibiting endogenous miR-375 by using miR-375 inhibitor (**D**). AEG-1 siRNA decreased AEG-1 mRNA expression (**E**) in CC cells. Error bars represent mean + s.d. and *P-*Values are represented as ****P<*0.001, *****P<*0.0001compared to the corresponding controls.

**Supplementary Figure. 3**

**
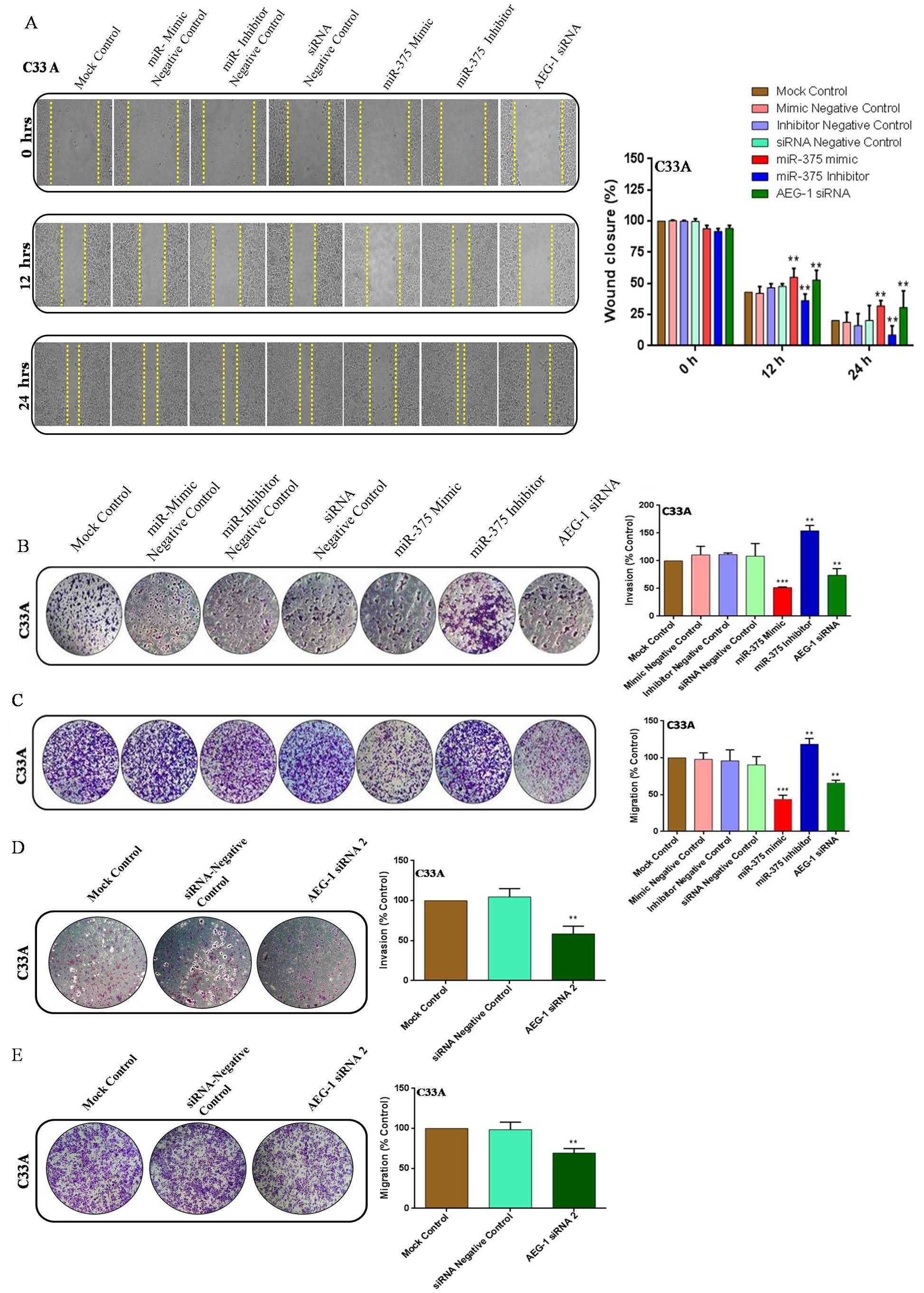
**

**S. Fig 3. Ectopic expression of miR-375 inhibits C33A.** *In vitro* scratch assay were performed in C33A cells at 0, 12 and 24 h post-transfection with miR-375 mimic, miR-375 Inhibitor, AEG-1 siRNA and their controls (**A**). Gap distance of cells was quantified by using Image J. Transwell invasion assay with Matrigel was performed in miR-375 mimic, miR-375 Inhibitor, AEG-1 siRNA and their controls transfected C33A cells (**B**). Transwell migration assay without Matrigel was performed in miR-375 mimic, miR-375 Inhibitor,AEG-1 siRNA and their controls transfected C33A cells (**C**). Transwell invasion and migration assay was performed in AEG-1 siRNA 2 and their negative control transfected C33A cells (**D-E**).The scale bars represent 100 *µm.* Error bars represent mean + s.d. and *P-*Values are represented as ***P<*0.05, ****P<*0.001compared to the corresponding controls.

**Supplementary Figure. 4**


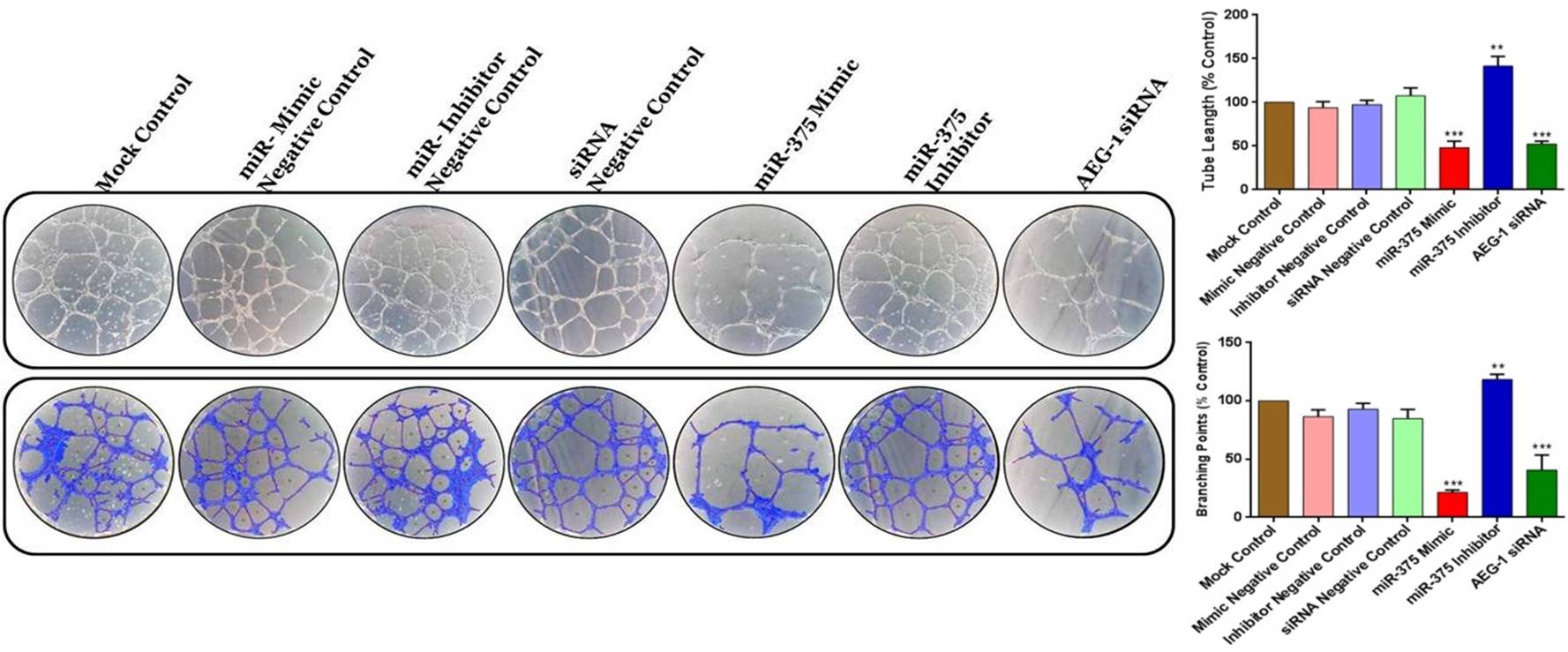


**S. Fig 4. miR-375 suppressed angiogenesis in HUVECs .**HUVECs were transfected with miR-375 mimic, miR-375 Inhibitor, AEG-1 siRNA and their controls and then cultured for 16 h incubation. The HUVECs capillary-like tubular structures were examined by branch points and total tube length. The scale bars represent 100 *µm.* Error bars represent mean + s.d. and *P-*Values are represented as ***P<*0.05, ****P<*0.001compared to the corresponding controls.

**Supplementary Figure. 5**


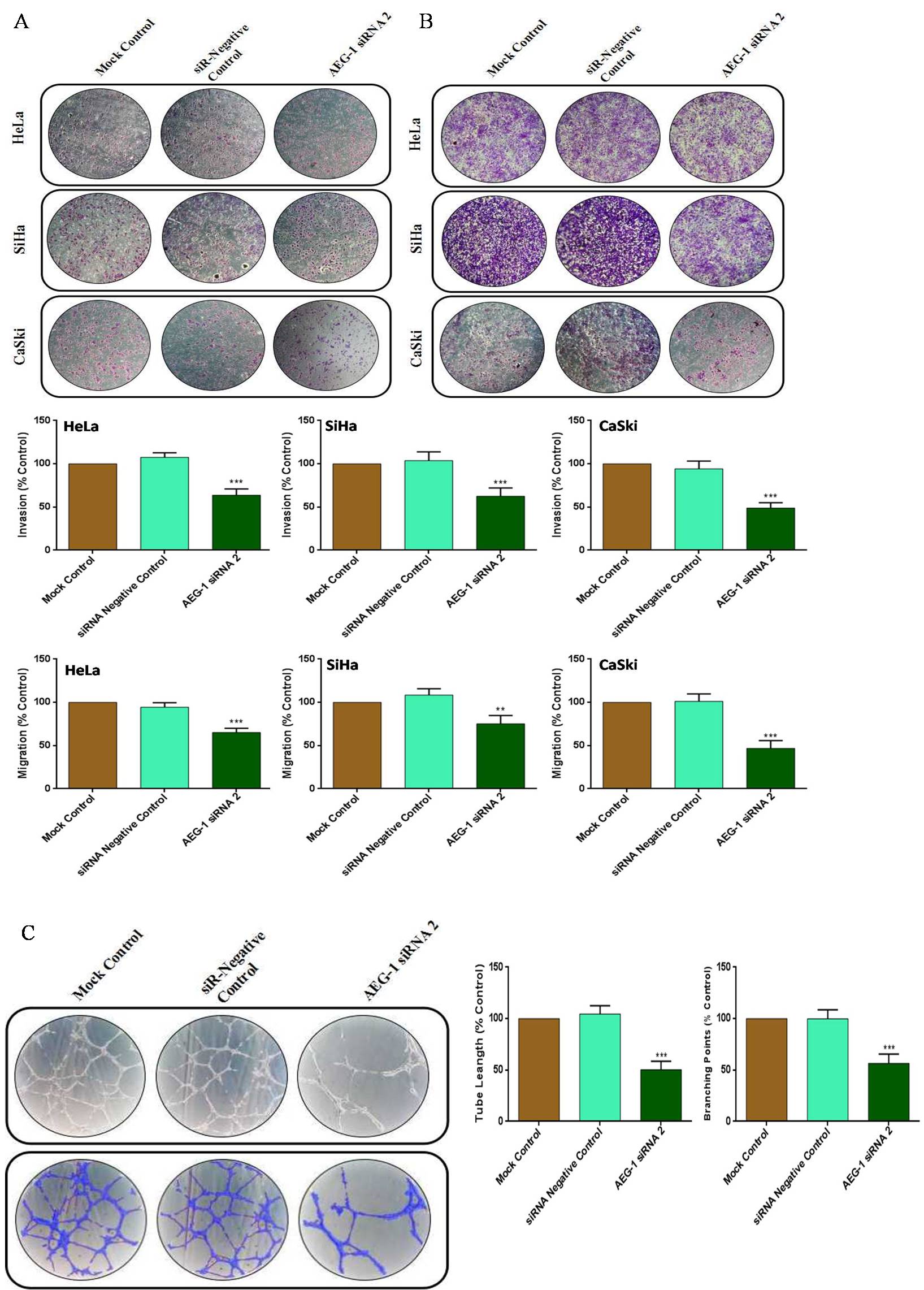


**S. Fig 5. AEG-1 siRNA 2 suppressed the invasion, migration and angiogenesis of cervical cancer cells.** Transwell invasion assay with Matrigel was performed in AEG-1 siRNA 2 and their controls transfected CC cells (**A**). Transwell migration assay without Matrigel was performed in AEG-1 siRNA 2 and their controls transfected CC cells (**B**). HUVECs were transfected with AEG-1 siRNA 2 and their controls and then cultured for 16 h incubation. The HUVECs capillary-like tubular structures were examined by branch points and total tube length (**C**). The scale bars represent 100 *µm.* The scale bars represent 100 *µm.* Error bars represent mean + s.d. and *P-*Values are represented as ***P<*0.05, ****P<*0.001compared to the corresponding controls.

**Supplementary Figure. 6**

**
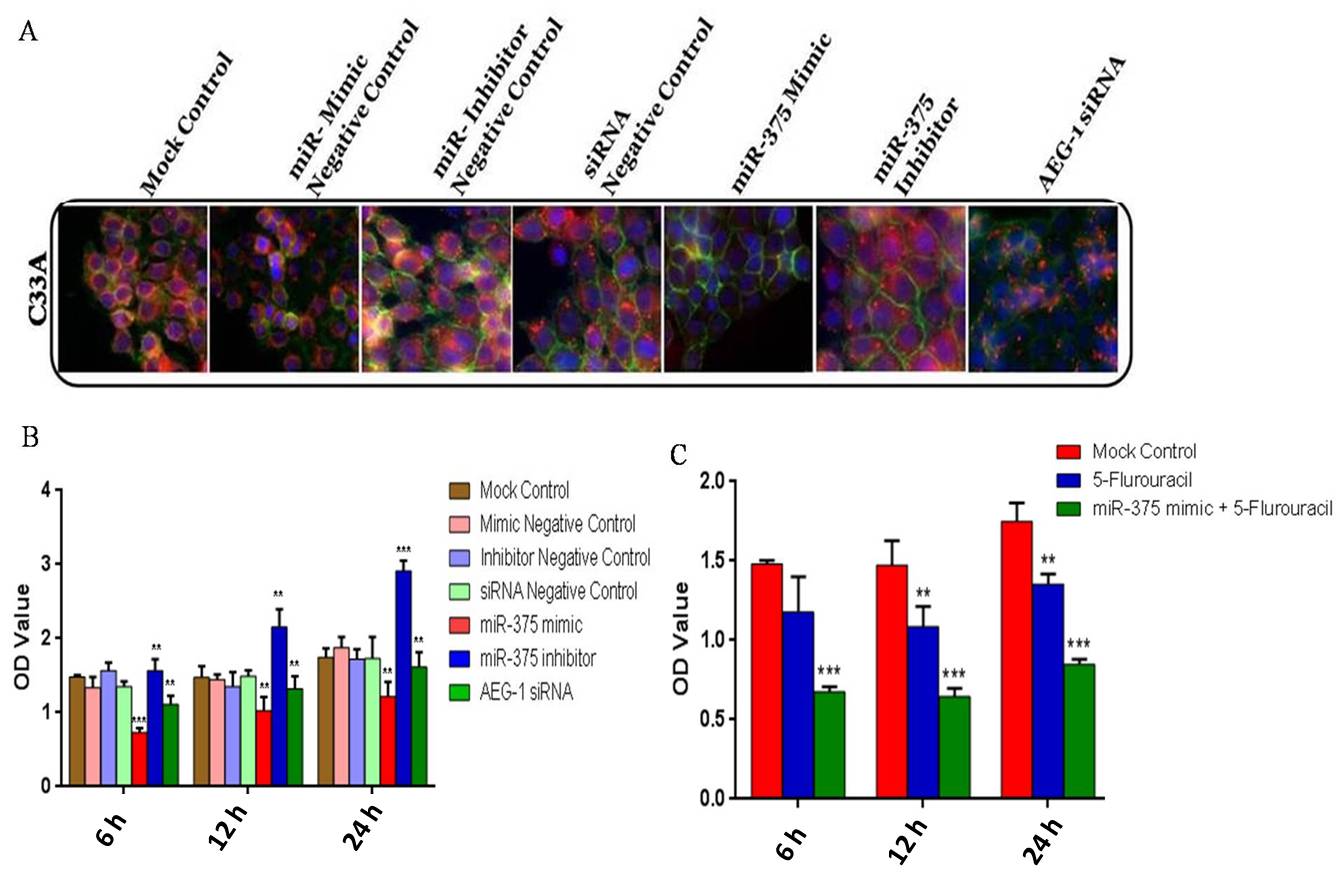
**

**S. Fig 6. miR-375 inhibits cellular proliferation, enhance 5-Fluorouracil sensitivity and Immunocytochemistry in C33A.** AEG-1 Protein expression was determined by Immunocytochemistry (**A**).Effect of miR-375 mimic on C33A cells proliferation of by MTT assay (**B**). C33A cells were transfected with a mock control, 5-Fluorouracil and then the miR-375 mimic transfected cells were treated with IC_50_ Value of 5-Fluorouracil and incubated at different time intervals (6, 12 and 24 h) (**C**). The scale bars represent 100 *µm.* Error bars represent mean + s.d. and *P-*Values are represented as ***P<*0.05, ****P<*0.001compared to the corresponding controls.

**Supplementary Figure. 7**

**
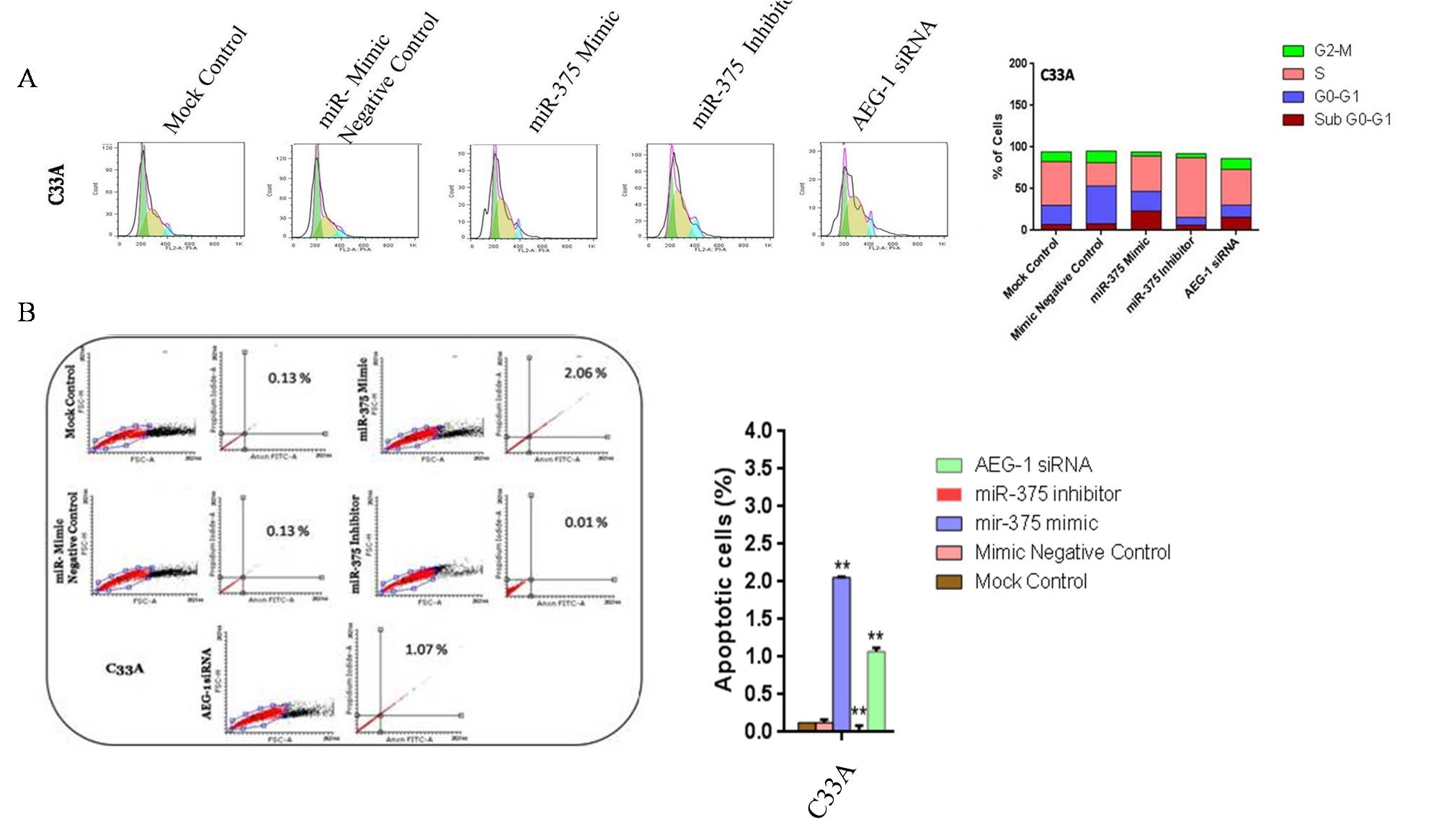
**

**S. Fig7. miR-375 induces cell cycle arrest and apoptosis *in vitro*.** C33A cells were transfected with miR-375 mimic, miR-375 Inhibitor,AEG-1 siRNA and their controls and 48h later, the effect of miR-375 on cell cycle was evaluated by the Flow cytometry (**A**). Apoptosis was assessed in cells transfected with miR-375 mimic, miR-375 Inhibitor, AEG-1 siRNA and their controls by Flow cytometry (**B**). Error bars represent mean + s.d. and *P-*Values are represented as ***P<*0.05 compared to the corresponding controls.

**Supplementary Figure. 8**

**
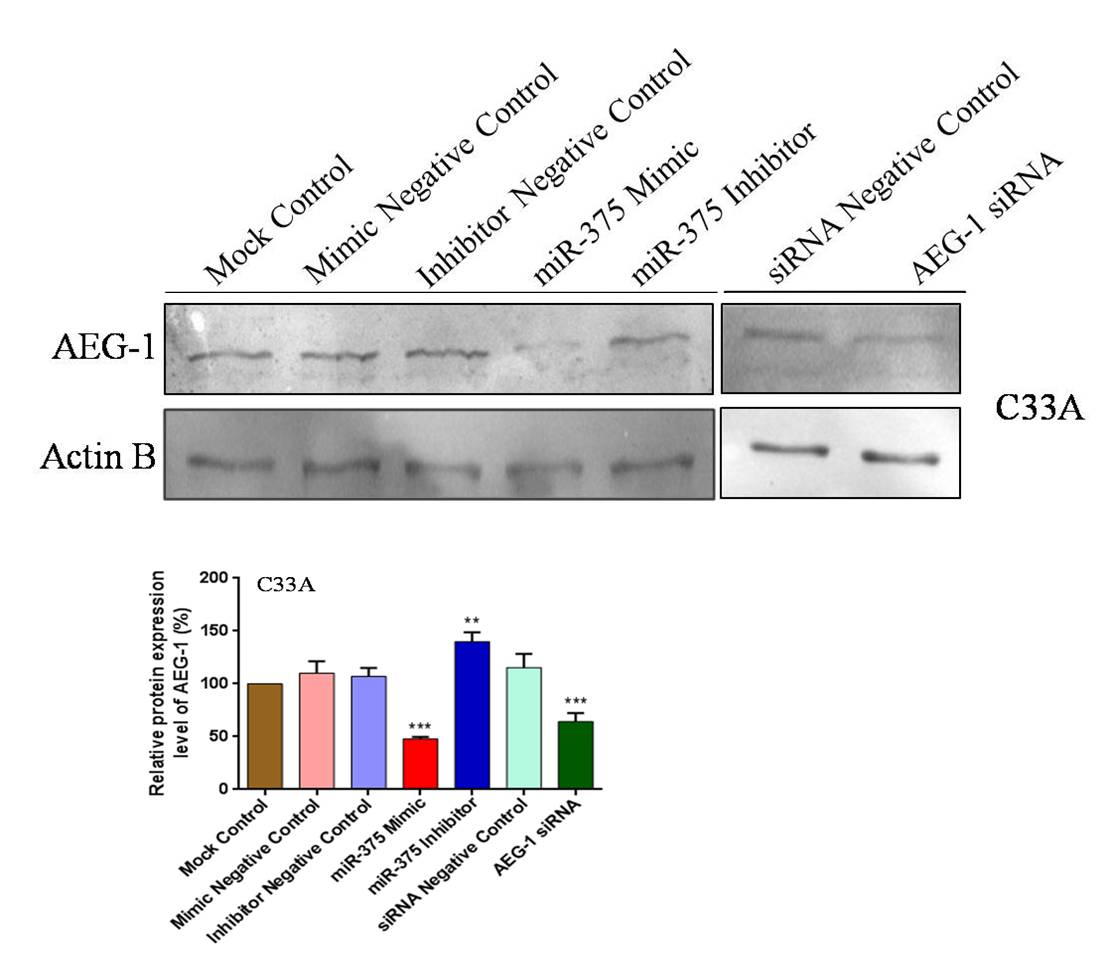
**

**S. Fig 8. miR-375 suppresses AEG-1 protein level in C33A.**Western blot validation of AEG-1 protein expression in C33A cells after transfecting miR-375 mimic, miR-375 Inhibitor, AEG-1 siRNA and their controls with β-actin as the loading control .Error bars represent mean + s.d. and *P-*Values are represented as ***P<*0.05, ****P<* 0.001 compared to the corresponding controls.

**Supplementry Fig . 9:**


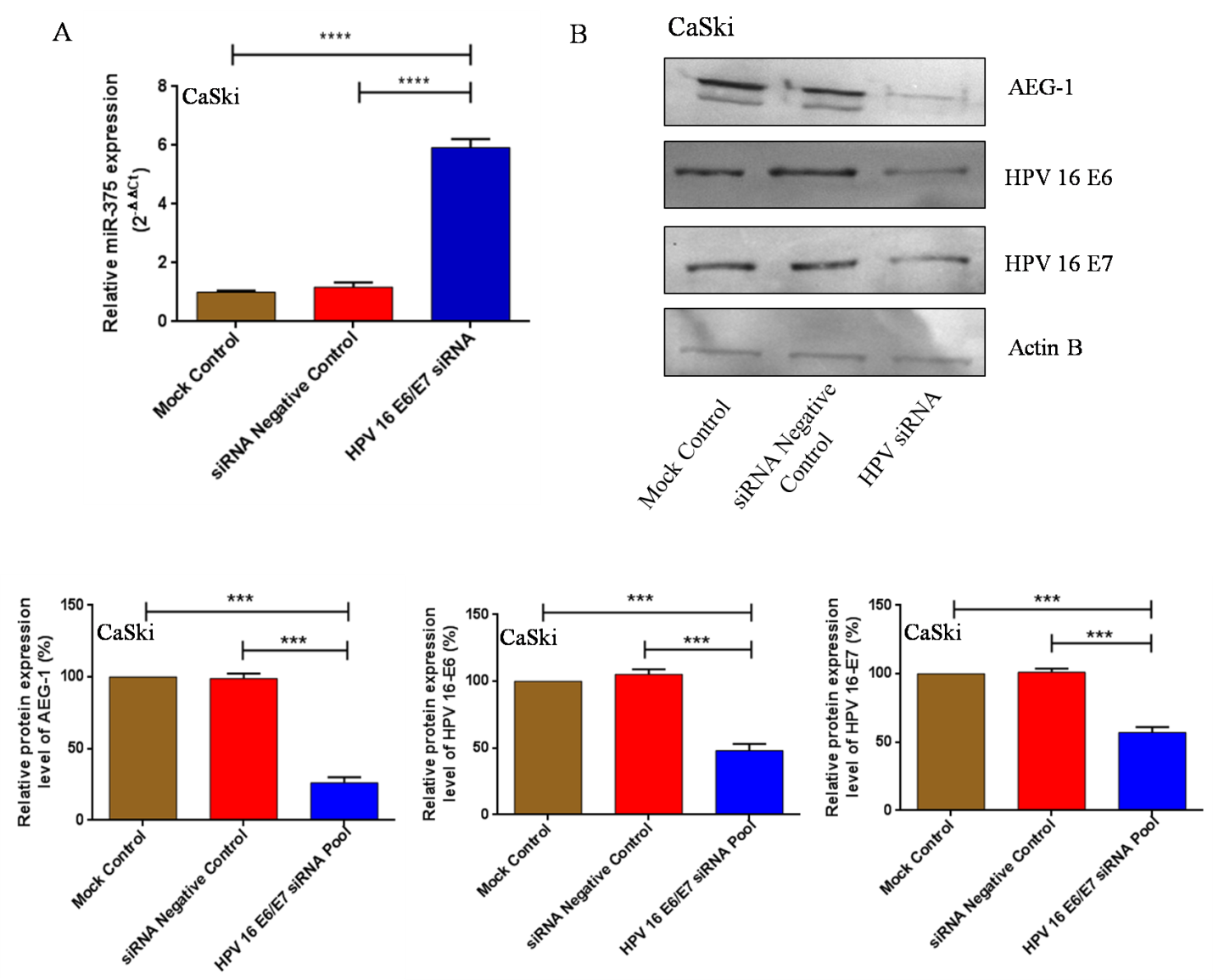


**S. Fig 9. HPV 16 E6/E7 regulates miR-375 and AEG-1 expression in CaSki cell line:** Effect of HPV16 E6/E7 siRNA on miR-375 expression (A) and AEG-1 expression (B). Error bars represent mean + s.d. and *P-*Values are represented as ****P<*0.001, *****P<*0.0001 compared to the corresponding controls.

**Supplementary Table. 1:**

**miR-375 and U6 Reverse Transcription (RT) Primer**

| **S.No** | **Gene** | **RT** |
| --- | --- | --- |
| **1** | miR-375 | 5’-GTCGTATCCAGTGCAGGGTCCGAGGTATTCGCACTGGATACGACTCACGC-3’ |
| **2** | U6 | 5’-AACGCTTCACGAATTTGCGT-3’ |

**Supplementary Table.2:**

**List of qRT-PCR Primers**

| **S.No** | **Gene** | **Forward Primer** | **Reverse Primer** | **Length**  **(bp)** | **GC ( %)** | **Melting**  **Temperature**  **(^0^C)** |
| --- | --- | --- | --- | --- | --- | --- |
| 1. | miR-375 | 5’-AGCCGTTTGTTCGTTCGGCT-3’ | 5’-GTGCAGGGTCCGAGGT-3’ | 86 | 59 | 78.29 |
| 2 | U6 | 5’-CTCGCTTCGGCAGCACA-3’ | 5’-AACGCTTCACGAATTTGCGT-3’ | 94 | 47 | 76.80 |
| 3 | AEG-1 | 5’-AAATAGCCAGCCTATCAAGACTC-3’ | 5’-TTCAGACTTGGTCTGTGAAGGAG-3’ | 178 | 43 | 73.68 |
| 4. | GAPDH | 5’-ATGGGGAAGGTGAAGGTCG-3’ | 5’-GGGTCATTGATGGCAACAATATC-3’ | 107 | 51 | 78.00 |
| 5 | HPV 16 E6 | 5’-GCAAAGACATCTGGACAAAAAG-3’ | 5’-ACCGACCCCTTATATTATGGAATC-3’ | 50 | 40 | 69.22 |
| 6. | HPV 16 E7 | 5’-GAACCGGACAGAGCCCATTA-3’ | 5’-ACACTTGCAACAAAAGGTTACA-3’ | 48 | 42 | 68.47 |
| 7. | HPV 18 E6 | 5’-GATCTGTGCACGGAACTGAACA-3’ | 5’-GGTTATTTCTATGTCTTGCAGTG-3’ | 48 | 44 | 70.41 |
| 8. | HPV 18 E7 | 5’-CAACGTCACACAATGTTGTGTA-3’ | 5’-TCAATTCTGGCTTCACACTTAC-3’ | 50 | 40 | 70.11 |
